# Two distinct regions in micellar aggregates identified with pyrene-labeled dendrimers

**DOI:** 10.1126/sciadv.aed4545

**Published:** 2026-07-08

**Authors:** Donghan Liu, Sanjay Patel, Jean Duhamel

**Affiliations:** Institute for Polymer Research, Waterloo Institute for Nanotechnology, Department of Chemistry, University of Waterloo, 200 University Avenue West, Waterloo, N2L 3G1, Canada.

## Abstract

This study challenges the basic representation that micellar aggregates (MAs) are simple assemblies of ionic surfactants held together by their hydrophobic tails and stabilized by their charged headgroups by demonstrating that they exhibit specific surfactant microdomains. These microdomains are the result of local phase separation between charged and partially neutralized with sodium chloride surfactants inside the same MA of SDS. They were characterized with pyrene-labeled polyamidoamine dendrimers. The charged and partially neutralized surfactants were found to constitute the polar edge and apolar middle regions of MA, respectively, among which the dendrimers would spatially partition themselves. The fluorescence response of the dendrimers was directly related to the volume fraction of the edge region calculated by assuming that the MAs were cylinders whose edges were capped with two identical hemispheres consisting of 35 charged SDS molecules. The fact that merging the two hemispheres generates a spherical SDS micelle led to a general mechanism rationalizing the formation of MA.

## Teaser

Phase separation between charged and neutralized surfactants identified by pyrene excimer formation in micellar aggregates.

## INTRODUCTION

**Fig. 1.**
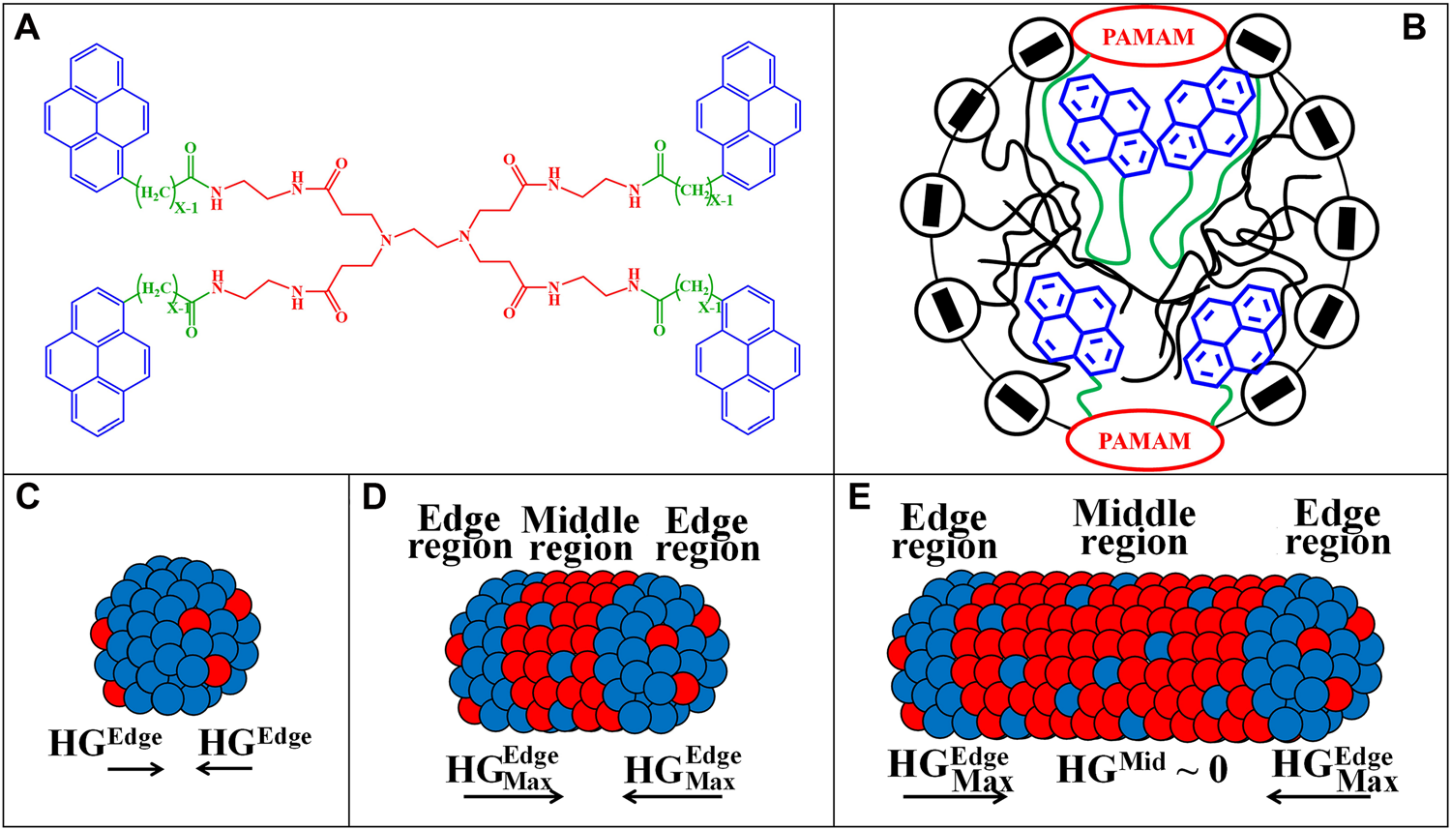
Schematic representation of dendrimers, MAs, and their interactions. Top row: (**A**) Chemical structure of the PyCX-PAMAM-G0 dendrimers with *X* = 4, 6, 8, 10, and 12 and (**B**) illustration of a PyCX-PAMAM-G0 sample in an SDS micelle (top; *X* = 10 and 12) undergoing and (bottom; *X* = 4, 6, and 8) not undergoing a conformational inversion. Bottom row: Depiction of the spatial arrangement of the (blue) charged and (red) partially neutralized SDS molecules in the (**C**) SDS micelles, (**D**) elongated NSMA, and (**E**) extended rod-like NSMA.

Lipids are the ultimate surfactants. They are involved in a variety of cellular functions that include cell signaling, intracellular molecular trafficking, or vesicular formation for endo- or exocytosis in eukaryote cells (*1*–*9*). Lipids achieve some of these tasks by phase separating into lipid rafts to generate vesicles that are loaded with small molecules like cholesterol or lipids, proteins, or genetic material enabling molecular trafficking between intracellular membranous organelles (*3*, *5*, *9*). Despite the recognized importance of the well-documented surfactant phases (*10*) in drug delivery (*11*–*13*), local surfactant-surfactant phase separation in micellar aggregates (MAs) is hardly documented in contrast to lipid-lipid phase separation in lipid membranes (*14*–*16*). The lack of information about surfactant microdomains (SMs) generated in MA stems, in part, from their small sizes. Compared to lipid rafts that might span hundreds of nanometers in micrometer-sized lipid membranes (*3*, *5*), SM can only be a few nanometers in size to fit within a rod- or disk-like MA that might be up to a few tens of nanometers long or wide, respectively (*17*–*22*). Such small dimensions generated by highly mobile surfactants inside the fluid interior of MA make it challenging to characterize SM by traditional experimental methods. For instance, scattering techniques like small-angle neutron scattering (SANS) or dynamic light scattering (DLS) have been used extensively to determine the overall size and shape of MA (*17*–*21*) but cannot probe minute compositional differences between the SM of MA, particularly so if the SMs are generated by different forms of a same surfactant. The nature of the MA interior is typically probed with hydrophobic fluorescent molecular probes (FmPs) that respond to polarity differences (*23*–*25*). Unfortunately, FmPs fail to distinguish between different SM in an MA because they drift toward the region of the MA with which they share the strongest affinity. Contrary to FmP, fluorescent macromolecular probes (FMPs) like generation-0 pyrene-labeled polyamidoamine (PAMAM) dendrimers (PyCX-PAMAM-G0; Fig. 1A) do not drift in an MA and report on the entire MA interior (*26*–*28*). Although fluorescence resonance energy transfer (FRET) is typically viewed as the go to fluorescence technique to probe macromolecular conformations, it is poorly suited to study macromolecules like the PAMAM dendrimers, whose complex architectures prevent their labeling with a single donor and a single acceptor. Labeling the terminals of dendrimers with multiple donors and acceptors, where the dendrimer internal segments are highly mobile resulting in multiple time-dependent distances [*r*_DA_(*t*,*i*,*j*)] separating every donor (*i*) and acceptor (*j*) pairs, makes the quantitative analysis of the FRET signal challenging, as has been discussed earlier (*29*, *30*). These problems are eliminated for macromolecules labeled at two specific positions in solution, where only one time-dependent distance [*r*_DA_(*t*,1,1)] needs to be considered (*31*–*33*), or for macromolecules embedded in a rigid polymer matrix, where *r*_DA_(*i*,*j*) is now time independent (*34*–*39*). In such cases, FRET has proved to be a most effective technique to probe their macromolecular properties. In the case of the PyCX-PAMAM-G0 dendrimers, however, the direct correspondence existing between the average rate constant (<*k*>) for excimer formation between an excited and a ground-state pyrene covalently attached to a macromolecule and the local pyrene concentration ([*Py*]_loc_) inside the macromolecular volume makes pyrene excimer formation (PEF) the go to fluorescence technique to probe macromolecular conformations in solution (*26*–*28*, *40*). Consequently, this report illustrates how PyCX-PAMAM-G0 dendrimers can be used as FMP to characterize quantitatively the size of SM coexisting inside an MA generated by SDS in aqueous NaCl solutions.

### Conformation of pyrene-labeled PAMAM dendrimers in MAs

Given the low solubility of pyrene in water (0.7 μM) (*41*), a PAMAM-G0 dendrimer bearing four 1-pyrenealkanoyl groups is insoluble in water despite its hydrophilic PAMAM core. However, the PyCX-PAMAM-G0 dendrimers can be incorporated inside MA. Studies have established that the polar PAMAM core sits at the micellar interface with water (*42*–*45*) and the dye pyrene locates itself in the palisade of surfactant micelles (*46*–*50*). Linking the PAMAM-G0 core to pyrene with alkanoyl linkers increasing in length from butanoyl (*X* = 4) to hexanoyl (*X* = 6), octanoyl (*X* = 8), decanoyl (*X* = 10), and dodecanoyl (*X* = 12) enables one to progressively promote hydrophobic interactions between the linker and the micellar interior (Fig. 1, A and B). The shorter linkers with *X* = 4, 6, and 8 have been found to not interact with the hydrophobic MA interior in contrast to the longer linkers with *X* = 10 and 12 (*27*, *28*). These hydrophobic interactions lead to an increase in the local pyrene concentration ([*Py*]_loc_) inside the dendrimers with the longer linkers, which is experimentally detected from an increase in the average rate constant (<*k*>) of intramolecular PEF between an excited and a ground-state pyrenyl label in a same PyCX-PAMAM-G0 sample (*26*–*28*, *40*). The increase in [*Py*]_loc_ for the PyC10- and PyC12-PAMAM-G0 dendrimers reflects the hydrophobicity gradient (HG) induced between the polar MA surface, where the PAMAM-G0 dendrimer sits, and the hydrophobic interior of the MA, which the longer 1-pyrenealkanoyl derivatives interact with (*27*, *28*). To date, the increase in [*Py*]_loc_ has been observed in pure SDS or pure dodecyltrimethylammonium bromide (DTAB) micelles (*27*) and, in MA, generated from mixtures of SDS and DTAB (*28*). Thus, the <*k*>-versus-[*Py*]_loc_ relationship (*40*) observed with PyCX-PAMAM-G0 dendrimers enables the characterization of the HG found in MA (*27*, *28*).

### Edges of SDS aggregates in NaCl aqueous solutions

SDS is probably the most studied surfactant (*51*–*54*) due to its widespread use in numerous applications (*51*–*53*) and the expectation that the properties identified for SDS aggregates are likely to also apply to other surfactant systems. Extensive studies by scattering techniques (small-angle x-ray scattering, SANS, and DLS), time-resolved fluorescence quenching, and less successfully cryo–transmission electron microscopy all concluded that, in aqueous solution, SDS forms small spherical micelles that morph into much larger nonspherical MA (NSMA) with high [NaCl] (*17*–*22*, *55*–*58*). The formation of NSMA is rationalized with the critical packing parameter (*cpp*) (*59*). Adding NaCl decreases electrostatic repulsions between the SDS headgroups resulting in a larger *cpp*, a lower curvature at the micellar surface, and larger MA that are no longer spherical (*17*–*22*, *55*–*58*). The departure from spherical micelles stems from the requirement that at least one dimension of the NSMA be constant and equal to about twice the surfactant tail length to ensure that the tails remain in contact across the diameter of a cylinder or the thickness of a membrane bilayer. The constraint that the MA be nonspherical with a region of low surface curvature also imposes that the NSMA edges be capped by surfactant structures having a much larger surface curvature to prevent exposure to water of the hydrophobic surfactant tails at the edges. This condition can only be met if the surfactants at the edges of the NSMA have a small *cpp*, imposing that the surfactant headgroups be charged and strongly repel each other. This logical conclusion dictates that surfactants in NSMA phase separate into a hydrophobic middle region constituted of surfactants with partially neutralized and densely packed headgroups and a relatively polar edge region constituted of charged surfactants enabling the electrostatic stabilization of the NSMA. The difference in polarity between the edge and middle regions of NSMA must induce a HG in the NSMA as illustrated in Fig. 1 (C to E) for the formation of rod-like NSMA. The existence of two regions with different polarities inside a same NSMA should promote interactions with amphiphilic molecules like the PyCX-PAMAM-G0 dendrimers in the same manner that lipid rafts do. These important features should warrant the characterization of NSMA edges. Yet even their existence is never discussed in the scientific literature (*60*–*62*).

Compositional differences between different regions of MA are typically probed with hydrophobic FmP. Unfortunately, the dynamic nature of the surfactants in NSMA leads FmP to drift toward the MA region with which they share the highest affinity, thus preventing the characterization of the heterogeneous interior of MA (*23*–*25*, *63*–*65*). In contrast, PyCX-PAMAM-G0 dendrimers distribute themselves randomly throughout NSMA and respond to changes in HG (*27*, *28*), representing appealing FMP for sensing the HG generated at the NSMA edges produced by adding NaCl to SDS aqueous solutions. Using PyCX-PAMAM-G0 dendrimers as FMP to study NSMA departs from the pioneering studies conducted by Turro, Tomalia and colleagues (*66*–*68*) who characterized the interactions between PAMAM dendrimers and surfactants.

## RESULTS

### Fluorescence spectra analysis for the PyCX-PAMAM-G0 dendrimers

**Fig. 2.**
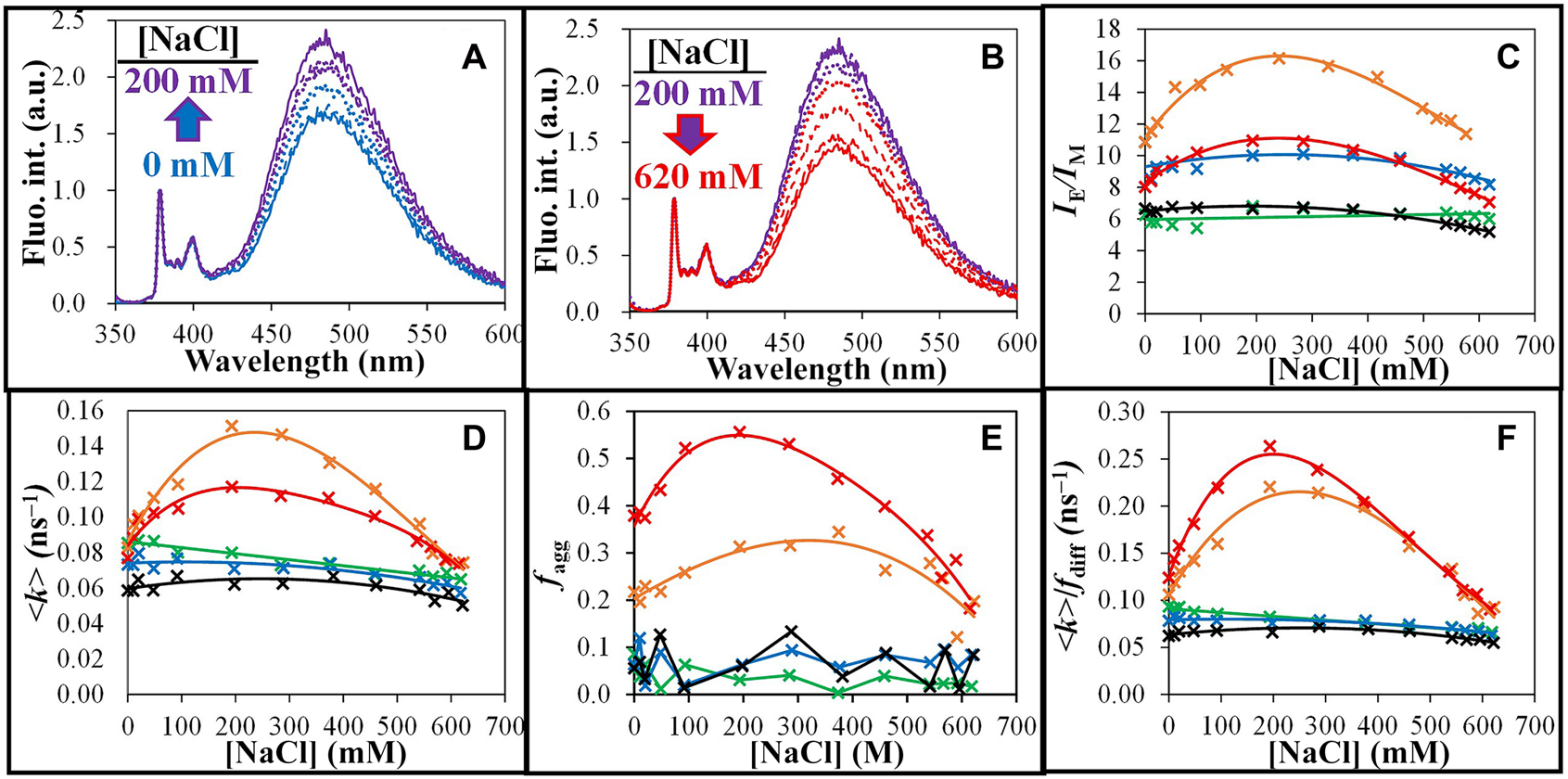
Analysis of fluorescence data. Fluorescence spectra normalized at the 0-0 transition of pyrene for (**A**) PyC12-PAMAM-G0 for [NaCl] increasing from (bottom) 0 to (top) 200 mM; (**B**) PyC12-PAMAM-G0 for [NaCl] increasing from (top) 200 to (bottom) 620 mM; and plots of (**C**) *I*_E_/*I*_M_, (**D**) <*k*>, (**E**) *f*_agg_, and (**F**) <*k*>/*f*_diff_ as a function of [NaCl] for (green) PyC4-, (blue) PyC6-, (black) PyC8-, (orange) PyC10-, and (red) PyC12-PAMAM-G0. [SDS] = 50 mM, [Py] = 2.4 μM, λ_ex_ = 344 nm. Fluo. int., fluorescence intensity; a.u., arbitrary units.

The synthesis and chemical characterization of the PyCX-PAMAM-G0 samples were described in earlier publications (*26*, *27*). The preparation of 50 mM SDS aqueous solutions with submicromolar PyCX-PAMAM-G0 concentration and instrumentation is described in the Supplementary Materials. Submicromolar PyCX-PAMAM-G0 concentrations ensured the solubilization of the dendrimers and that the probability of incorporating two or more dendrimers per NSMA was negligible (*27*, *28*). The fluorescence spectra of the PyCX-PAMAM-G0 dendrimers were acquired at different [NaCl] and are shown in fig. S5. The fluorescence spectra of the PyC12-PAMAM-G0 dendrimers normalized at 379 nm are shown in Fig. 2 (A and B). The pyrene monomer emits with sharp bands between 370 and 410 nm, and the excimer fluoresces with a broad structureless emission centered around 480 nm. PEF for PyC12-PAMAM-G0 increased in Fig. 2A with [NaCl] increasing up to 200 mM before decreasing in Fig. 2B for [NaCl] increasing from 200 to 620 mM. These effects were summarized in Fig. 2C where the fluorescence intensity ratio (*I*_E_/*I*_M_) of the excimer to the monomer was plotted as a function of [NaCl] for all the PyCX-PAMAM-G0 dendrimers.

The *I*_E_/*I*_M_ ratios cannot be directly compared from one sample to another due to the presence of residual unattached pyrene derivative, which affects their magnitude (*26*–*28*). Nevertheless, the *I*_E_/*I*_M_ ratio obtained for one pyrene-labeled macromolecule (PyLM) is informative as the residual amount of unattached pyrene derivative remains constant and *I*_E_/*I*_M_ reflects how [*Py*]_loc_ varies as a function of [NaCl] for a given PyCX-PAMAM-G0 sample (*69*).

The *I*_E_/*I*_M_ ratio showed little variation over the [NaCl] range of 0 to 620 mM for the PyCX-PAMAM-G0 samples with *X* = 4, 6, and 8, indicating little change in [*Py*]_loc_. This was expected because these dendrimers adopt their ideal conformation in surfactant micelles and are, therefore, insensitive to a change in HG (*27*, *28*). In contrast, the *I*_E_/*I*_M_-versus-[NaCl] profiles showed significant changes for the PyC10- and PyC12-PAMAM-G0 samples. Because a change in *I*_E_/*I*_M_ and thus [*Py*]_loc_ reflects a change in HG, these trends indicate that the HG generated in the NSMA formed by SDS first increases and then decreases for [NaCl] increasing first from 0 to 200 mM and then from 200 to 620 mM, respectively. They also reflect the ability of the PyCX-PAMAM-G0 dendrimers to probe the HG inside NSMA. Of note is that the fluorescence properties of pyrene inside SDS MA have been found to be insensitive to NaCl concentration (*70*, *71*), which rules out any possible changes in the photophysical properties of the pyrenyl labels of the PyCX-PAMAM-G0 dendrimers induced by NaCl. For a more quantitative understanding, the global model-free analysis of the fluorescence decays acquired with the PyCX-PAMAM-G0 samples (*26*–*28*, *40*) was conducted next.

### Fluorescence decay analysis for the PyCX-PAMAM-G0 dendrimers

The model-free analysis (*72*) yielded the average rate constant (<*k*>) of PEF and the molar fractions *f*_diff_ and *f*_agg_ (=1 − *f*_diff_) of pyrenyl labels forming excimer by diffusive pyrene-pyrene encounters or direct excitation of pyrene aggregates, respectively (*26*–*28*). *f*_agg_ was negligible at all [NaCl] in Fig. 2E for the PyCX-PAMAM-G0 samples with *X* = 4, 6, and 8 and <*k*> showed little variation for these samples in Fig. 2D. In contrast, both <*k*> and *f*_agg_ for the PyC10- and PyC12-PAMAM-G0 samples in Fig. 2 (D and E) passed through a maximum. The increase in <*k*> with [NaCl] increasing from 0 to 200 mM reflects an increase in [*Py*]_loc_, resulting in an increase in *f*_agg_ for the PyC10- and PyC12-PAMAM-G0 samples. This increase is remarkable as SDS forms larger NSMA, which should better accommodate the larger PyC10- and PyC12-PAMAM-G0 dendrimers. Instead, the observed increase in <*k*> and *f*_agg_ is driven by enhanced hydrophobic interactions between the longer alkanoyl linkers and the NSMA interior induced by an increased HG. Because the increase in *f*_agg_ reduces the number of pyrenyl labels available for PEF and, hence, <*k*>, the [<*k*>/*f*_diff_]^exp^ ratio was used instead to account for the changes in *f*_agg_ as done earlier (*73*, *74*). [<*k*>/*f*_diff_]^exp^ was plotted in Fig. 2F against [NaCl]. The PyCX-PAMAM-G0 samples with *X* = 4, 6, and 8 took low [<*k*>/*f*_diff_]^exp^ ratios, which varied little with [NaCl], whereas the PyCX-PAMAM-G0 samples with *X* = 10 and 12 showed a pronounced maximum centered at about 200 mM NaCl. The origin of the variations of [<*k*>/*f*_diff_]^exp^ with [NaCl] is discussed hereafter.

## DISCUSSION

### Interpretation of the [<*k*>/*f*_diff_]^exp^ trends

At low [NaCl], the increase in [<*k*>/*f*_diff_]^exp^ shown in Fig. 2F by the PyC10- and PyC12-PAMAM-G0 samples reflects a rise in the HG of the edge region (HG^Edge^) induced by the formation of the more hydrophobic middle region of the NSMA, as suggested in Fig. 1 (C to E). The increase in HG^Edge^ continues until [NaCl] reaches 200 mM, at which point HG^Edge^ takes its maximum HG^Edge,Max^ value, which remains constant for [NaCl] greater than 200 mM. As [NaCl] increases further up to 620 mM, the middle region continues growing while the edge region remains unchanged. Because the partial neutralization of the SDS headgroups in the middle region by NaCl results in a much smaller HG^Mid^, partitioning of the dendrimers between the expanding middle region and the edges results in the decreasing [<*k*>/*f*_diff_]^exp^-versus-[NaCl] trends observed for the PyC10- and PyC12-PAMAM-G0 dendrimers in Fig. 2F for [NaCl] increasing from 200 to 620 mM.

Partitioning of the PyC10- and PyC12-PAMAM-G0 dendrimers between the edge and middle regions suggests that [<*k*>/*f*_diff_]^exp^ should be related to the volume fraction (*f*_Edge_) of the edge region in the NSMA. *f*_Edge_ was obtained from Eq. 1, where *N*_agg_([NaCl] = 0 M) (~70) and *N*_agg_([NaCl]) are the aggregation numbers of an SDS micelle in pure water and of an NSMA formed by adding NaCl to an SDS aqueous solution, respectively. Implicit in the expression of *f*_Edge_ is the assumption that the number of charged surfactants in the edge region equals that of an SDS micelle in pure water. This assumption is inspired by the cylindrical NSMA (see Fig. 1, C to E) suggested in several studies (*17*–*22*, *55*–*58*) as merging the two hemispheres (edge region) capping the cylinder (middle region) would form a sphere equivalent to a pure SDS micelle. The function *N*_agg_([NaCl]) in Eq. 1 was obtained by parametrizing the *N*_agg_ values reported in the literature for SDS aqueous solutions at different [NaCl] (*58*, *70*) as described in the Supplementary Materials

$$f_{\text{Edge}}=\frac{N_{\text{agg}}\left(\left[\text{NaCl}\right]=0\;M\right)}{N_{\text{agg}}\left([\text{NaCl}]\right)}$$ (1)

**Fig. 3.**
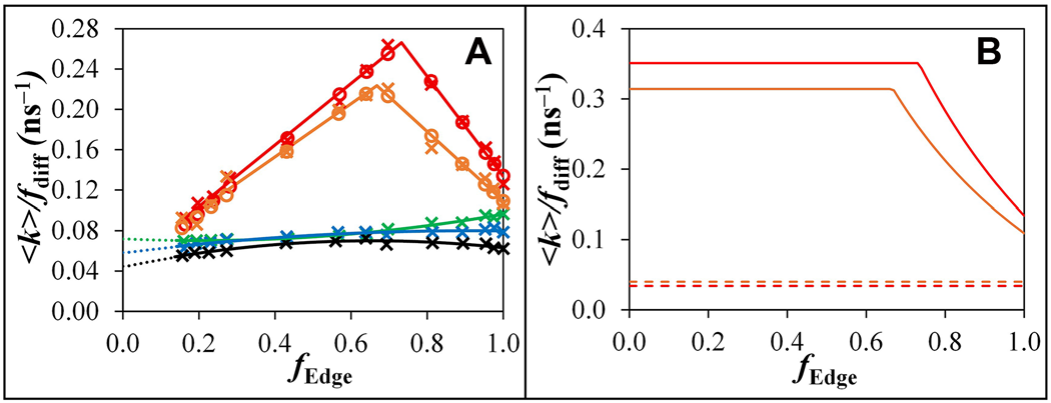
Interpretation of results according to the spatial partitioning theory. Plot of (**A**) (crosses) [<*k*>/*f*_diff_]^exp^ and (hollow circles) [<*k*>/*f*_diff_]^th^ as a function of *f*_Edge_ and (**B**) (solid line) [<*k*>/*f*_diff_]^Edge^ and (dashed line) [<*k*>/*f*_diff_]^Mid^ as a function of *f*_Edge_. Colors for the [<*k*>/*f*_diff_]^exp^ and [<*k*>/*f*_diff_]^th^ symbols: (green) PyC4-PAMAM-G0, (blue) PyC6-PAMAM-G0, (black) PyC8-PAMAM-G0, (orange) PyC10-PAMAM-G0, and (red) PyC12-PAMAM-G0. [SDS] = 50 mM, [Py] = 1.6 μM.

Plotting [<*k*>/*f*_diff_]^exp^ as a function of *f*_Edge_ in Fig. 3A perfectly linearized the trends in Fig. 2F demonstrating the importance of *f*_Edge_ in the interpretation of the [<*k*>/*f*_diff_]^exp^ trends. The trends with the PyC10- and PyC12-PAMAM-G0 dendrimers in Fig. 3A led to the identification of two clear-cut regimes. The regime corresponding to *f*_Edge_^Max^ ≤ *f*_Edge_ ≤ 1.0 reflects the buildup of HG^Edge^ as the middle region of the NSMA is becoming increasingly hydrophobic, and [<*k*>/*f*_diff_]^exp^ increases from its value ([<*k*>/*f*_diff_]^SDS^) found in SDS micelles at *f*_Edge_ = 1 to [<*k*>/*f*_diff_]^Edge,Max^ at *f*_Edge_ = *f*_Edge_^Max^. The second regime corresponding to 0 ≤ *f*_Edge_  ≤  *f*_Edge_^Max^ represents the partitioning of the PyC10- and PyC12-PAMAM-G0 dendrimers between the expanding middle region with a low [<*k*>/*f*_diff_]^Mid^ and the edge region with a constant [<*k*>/*f*_diff_]^Edge,Max^.

The partitioning of the PyC10- and PyC12-PAMAM-G0 dendrimers between the middle and edge regions led to Eq. 2, which was derived in the Supplementary Materials. Equation 2 provides a theoretical expression ([<*k*>/*f*_diff_]^th^) for the experimental ([<*k*>/*f*_diff_]^exp^) based on [<*k*>/*f*_diff_]^Edge^ and [<*k*>/*f*_diff_]^Mid^ representing the [<*k*>/*f*_diff_] ratio in the edge and middle regions, respectively. [<*k*>/*f*_diff_]^Edge^ is a function of *f*_Edge_, and [<*k*>/*f*_diff_]^Mid^ is a constant as shown in Fig. 3B, with [<*k*>/*f*_diff_]^Edge^ increasing sharply with decreasing *f*_Edge_ for *f*_Edge_^Max^ ≤ *f*_Edge_ ≤ 1.0 before remaining constant and equal to [<*k*>/*f*_diff_]^Edge,Max^ for 0 ≤ *f*_Edge_ ≤ *f*_Edge_^Max^. In contrast, [<*k*>/*f*_diff_]^Mid^ took a small and constant value for all *f*_Edge_ value.

The excellent agreement between the experimental [<*k*>/*f*_diff_]^exp^ and theoretical [<*k*>/*f*_diff_]^th^ ratios in Fig. 3A validates Eqs. 1 and 2 and leads to three key conclusions. First, NSMAs are compartmentalized into the middle and edge regions. Second, the edge region comprises ~70 SDS molecules according to Eq. 1. Third, the dendrimers partition themselves randomly between the two regions of the NSMA. Together, these three conclusions constitute the spatial partitioning theory, as represented by Eq. 2 which was derived in the Supplementary Materials.

$$\begin{array}{c} {\left[<k>/f_{\text{diff}}\right]}^{\text{th}}={\left[<k>/f_{\text{diff}}\right]}^{\text{Mid}}+\left({\left[<k>/f_{\text{diff}}\right]}^{\text{Edge}}\left(f_{\text{Edge}}\right)\right.\\ \left.-{\left[<k>/f_{\text{diff}}\right]}^{\text{Mid}}\right)\times f_{\text{Edge}} \end{array}$$ (2)

### Mechanism for the formation of NSMA

**Fig. 4.**
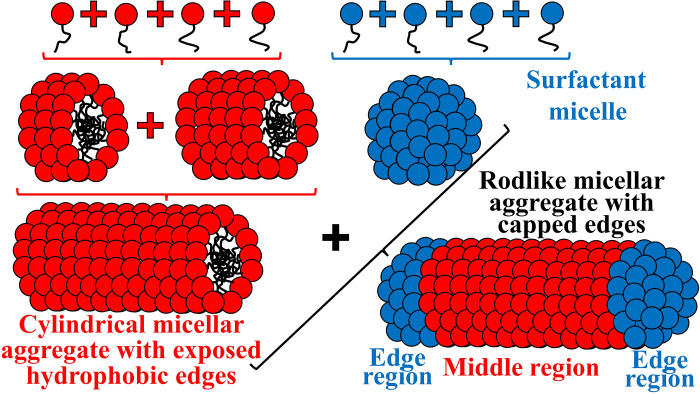
Mechanism for the formation of cylindrical NSMA. Encounters between a (blue) SDS micelle and a (red) hydrophobic MA with exposed hydrophobic edges yield NSMA.

The linear [<*k*>/*f*_diff_]^exp^-versus-*f*_Edge_ trends in Fig. 3A underline the importance of *f*_Edge_. As demonstrated earlier, the number of charged SDS molecules in the edge region of an NSMA always equals *N*_agg_([NaCl] = 0 M) (~70). This unexpectedly simple insight offers a basis for a straightforward mechanism describing the formation of NSMA. The partially neutralized SDS molecules being more hydrophobic than their nonneutralized counterparts should self-assemble first into large NSMA, while the remaining charged SDS molecules, which are subject to electrostatic repulsion, lead to a slower formation of SDS micelles. The large NSMA would have open edges exposing the hydrophobic dodecyl tails of the surfactants to water. Over time, diffusive encounters between NSMA and charged SDS micelles would lead to the formation of stabilized NSMA (sNSMA) with a same number of charged SDS molecules at their edges (see Fig. 4) where the charged micelle becomes the edge region and stabilizes the MA by capping the exposed alkyl tails. Increasing [NaCl] would increase the number of neutralized SDS molecules, which would associate into larger NSMA, and reduce the number of SDS micelles made of charged SDS available to cap the edges of the NSMA, resulting in the formation of larger sNSMA as observed experimentally.

This study established that surfactants phase separate into a polar edge and a hydrophobic middle region coexisting inside a same NSMA generated from aqueous solutions of SDS and NaCl. The edge region consists of ~70 charged SDS molecules that are separate from the middle region, which grows as a function of [NaCl]. Partitioning of the dendrimers between the edge and middle regions yields the linear [<*k*>/*f*_diff_]^exp^-versus-*f*_Edge_ trends in Fig. 3A, validating the proposed spatial partitioning theory describing the distribution of the PyCX-PAMAM-G0 dendrimers in NSMA and leading to the general mechanism in Fig. 4 for the formation of NSMA, such as those formed between SDS and DTAB (*28*).

## MATERALS AND METHODS

### Materials

The synthesis and characterization by ^1^H NMR and mass spectrometry of the PyCX-PAMAM-G0 samples with *X* = 4, 6, 8, 10, and 12 has been reported in earlier publications (*26*, *27*). Omnipur SDS (>99%) and sodium chloride (99%) were purchased from Sigma-Aldrich and used as received. All aqueous solutions were prepared with doubly distilled deionized water from Millipore Milli-RO 10 Plus and Milli-Q UF Plus (Bedford, MA).

### PyCX-PAMAM-G0 sample preparation

The procedure established in an earlier study was followed. The PyCX-PAMAM-G0 dendrimers and SDS were first suspended in chloroform, before evaporating the solvent under a gentle air stream resulting in a thin layer of white powder at the bottom of the round bottom flask. Upon rehydration with the appropriate amount of water, clear solutions of the PyCX-PAMAM-G0 samples were obtained in 50 mM SDS solutions. Because the 50 mM SDS concentration exceeded the 8 mM critical micelle concentration, SDS micelles formed that provided the hydrophobic environment required for the solvation of the 1-pyrenealkanoyl groups of the PyCX-PAMAM-G0 samples. Consequently, the solution exhibited well-defined absorption bands, suggesting that the pyrenyl groups were well solvated. Furthermore, the pyrene excimer fluorescence decays showed a defined rise time as expected for excimer formation through diffusive encounters between solvated pyrenyl moieties. The NaCl concentration in the 50 mM SDS aqueous solution was adjusted from 0 to 100 mM and from 100 to 620 mM by adding a minimum volume of a 1 or 5 M NaCl, respectively.

### Instrumentation

#### Ultraviolet-visible absorption measurements

To obtain a reasonable signal-to-noise ratio, the absorption spectra of dilute 0.2 × 10^−6^ M and 0.4 × 10^−6^ M PyCX-PAMAM-G0 aqueous solutions containing 50 mM SDS were measured using a 5.0-cm path-length quartz cell on a Cary 100 ultraviolet-visible spectrophotometer.

#### Steady-state fluorescence

The xenon arc lamp of a HORIBA QM-400 spectrofluorometer was used to excite the PyCX-PAMAM-G0 solutions at 344 nm. The fluorescence spectra of the PyCX-PAMAM-G0 solutions were acquired from 350 to 650 nm with a 1-nm slit width on the excitation and emission monochromators. Integration of the fluorescence spectra from 376 to 382 nm and from 500 to 530 nm yielded the fluorescence intensity of the pyrene monomer (*I*_M_) and excimer (*I*_E_), respectively. The *I*_E_/*I*_M_(SSF) ratio was obtained by dividing *I*_E_ by *I*_M_, which was taken as a measure of the PEF efficiency for a given PyCX-PAMAM-G0 solution.

#### Time-resolved fluorescence

A FluoroHub time-resolved fluorometer from HORIBA was used to obtain the pyrene monomer and excimer fluorescence decays of the PyCX-PAMAM-G0 aqueous solutions. The solutions were excited with a 336-nm DeltaDiode laser. Both pyrene monomer and excimer fluorescence decays had 20,000 counts at their decay maximum and were acquired at 379 and 510 nm over 1024 channels with a time-per-channel of 0.87 ns and a 370- and 495-nm cutoff filter, respectively. The cutoff filters were inserted at the entrance of the emission monochromator to prevent stray light from reaching the photomultiplier tube detector. A triangular aluminum monolith was used for the instrument response function, which was obtained by setting the emission wavelength at the same 336-nm excitation wavelength.

### Global model-free analysis of the monomer and excimer fluorescence decays

The fluorescence emitted by PyLMs is due to three pyrene species, whose behavior is well described by the MFA (*70*). They are the species *Py*_diff_*, *Py*_free_*, and *Py*_agg_*. *Py*_diff_* describes those pyrenyl labels (*Py*_diff_*) forming excimer by diffusive encounters. The species *Py*_free_* represents pyrenyl labels that are not attached to the macromolecule and emit with their lifetime (τ_M_). The species *Py*_agg_* represents the two preaggregated pyrene subspecies *E*0* and D* that are well and poorly stacked with a ground-state pyrene label and emit with a shorter and longer lifetimes τ_E0_ and τ_D_, respectively. The global MFA of the pyrene monomer and excimer fluorescence decays yields the molar fractions *f*_diff_, *f*_free_, *f*_E0_, and *f*_D_ of the pyrene species *Py*_diff_*, *Py*_free_*, *E*0*, and D*, respectively, with *f*_agg_ being equal to the sum *f*_E0_ + *f*_D_ representing the aggregated pyrenes (*Py*_agg_*).

The MFA acknowledges that any fluorescence decay can be fitted with a sum of exponentials. Consequently, the sum of exponentials shown in Eq. 3 is used to fit the pyrene monomer fluorescence decays. The front part of Eq. 3 describes PEF by diffusive encounters, whereas the exponential with the longest lifetime τ_M_ represents the species *Py*_free_*. Equation 4 was derived from the classic kinetic scheme for PEF and is used to the pyrene excimer fluorescence decays. The Marquardt-Levenberg algorithm (*75*) was used to optimize the parameters in Eqs. 3 and 4. These parameters were used to determine the molar fractions *f*_diff_ (=*f*_diffE0_ + *f*_diffD_), *f*_free_, and *f*_agg_ (=*f*_E0_ + *f*_D_), which represent the molar fractions of the pyrene species, which form excimer by diffusion, do not form excimer, and are aggregated and form excimer by direct excitation

$${\left[{\mathrm{Py}}^{*}\right]}_{\left(t\right)}={\left[{\mathrm{Py}}_{\text{diff}}^{*}\right]}_{\left(t=0\right)}\times \sum_{i=1}^{n}a_{i}\times \text{exp}\left(-t/\tau_{i}\right)+{\left[{\mathrm{Py}}_{\text{free}}^{*}\right]}_{\left(t=0\right)}\times \text{exp}\left(-t/\tau_{M}\right)$$ (3)

$$\begin{array}{c} {\left[E^{*}\right]}_{\left(t\right)}=-{\left[{\mathrm{Py}}_{\text{diff}}^{*}\right]}_{\left(t=0\right)}\times \sum_{i=1}^{n}a_{i}\frac{\frac{1}{\tau_{i}}-\frac{1}{\tau_{M}}}{\frac{1}{\tau_{i}}-\frac{1}{\tau_{E0}}}\;\text{exp}\left(-t/\tau_{i}\right)\\ +\left({\left[E0^{*}\right]}_{\left(t=0\right)}+{\left[{\mathrm{Py}}_{\text{diff}}^{*}\right]}_{\left(t=0\right)}\times \sum_{i=1}^{n}a_{i}\frac{\frac{1}{\tau_{i}}-\frac{1}{\tau_{M}}}{\frac{1}{\tau_{i}}-\frac{1}{\tau_{E0}}}\right)\times \text{exp}\left(-t/\tau_{E0}\right)\\ -{\left[{\mathrm{Py}}_{\text{diff}}^{*}\right]}_{\left(t=0\right)}\times \sum_{i=1}^{n}a_{i}\frac{\frac{1}{\tau_{i}}-\frac{1}{\tau_{M}}}{\frac{1}{\tau_{i}}-\frac{1}{\tau_{D}}}\;\text{exp}\left(-t/\tau_{i}\right)\\ +\left({\left[D^{*}\right]}_{\left(t=0\right)}+{\left[{\mathrm{Py}}_{\text{diff}}^{*}\right]}_{\left(t=0\right)}\times \sum_{i=1}^{n}a_{i}\frac{\frac{1}{\tau_{i}}-\frac{1}{\tau_{M}}}{\frac{1}{\tau_{i}}-\frac{1}{\tau_{D}}}\right)\times \text{exp}\left(-t/\tau_{D}\right)\\ +{\left[{\mathrm{ES}}^{*}\right]}_{\left(t=0\right)}\times \text{exp}\left(-t/\tau_{\text{ES}}\right) \end{array}$$ (4)

The preexponential factors (*a*_i_) and decay times (τ_i_) obtained from the MFA of the fluorescence decays in the front part of Eq. 3 were used to calculate the average lifetime <τ> with Eq. 5. <τ> could then be used to determine <*k*> according to Eq. 6

$$<\tau >=\frac{\sum_{i=1}^{n}a_{i}\tau_{i}}{\sum_{i=1}^{n}a_{i}}$$ (5)

$$<k>=\frac{1}{<\tau >}-\frac{1}{\tau_{M}}$$ (6)

In the global MFA of the pyrene monomer and excimer fluorescence decays, the instrument response function was convoluted with Eqs. 3 and 4, and both convolution products were compared with the experimental decays after optimization of the MFA parameters according to the Marquardt-Levenberg algorithm (*28*). A fit was deemed satisfactory if the global χ^2^ was smaller than 1.3, and the residuals and autocorrelation of the residuals were randomly distributed around zero.
