# Supplementary material for: Two distinct regions in micellar aggregates identified with pyrene-labeled dendrimers
Source: Sci Adv. 2026 Jul 8;12(28):eaed4545. doi: 10.1126/sciadv.aed4545 (PMC13344352; doi:10.1126/sciadv.aed4545)
Supplement: Supplementary file 1 — Supplementary Text Figs. S1 to S5 Tables S1 to S4 Legends for data S1 to S3 [file sciadv.aed4545_sm.pdf]

Science Advances
AAAS

Supplementary Materials for

Two distinct regions in micellar aggregates identified with pyrene-labeled dendrimers

Donghan Liu *et al.*

Corresponding author: Jean Duhamel, jduhamel@uwaterloo.ca

Sci. Adv. **12**, eaed4545 (2026)
DOI: 10.1126/sciadv.aed4545

The PDF file includes:

Supplementary Text
Figs. S1 to S5
Tables S1 to S4
Legends for data S1 to S3

Other Supplementary Material for this manuscript includes the following:

Data S1 to S3

Supplementary Text

Quantitative analysis of the $[\langle k\rangle/f_{\mathrm{diff}}]^{\mathrm{exp}}$-*vs.*-$f_{\mathrm{Edge}}$ trends

In Eq. 1, $[\langle k\rangle/f_{\mathrm{diff}}]^{\mathrm{Mid}}$ is constant and its value is given in Table S1 whereas $[\langle k\rangle/f_{\mathrm{diff}}]^{\mathrm{Edge}}$ is a function of f_{Edge}, that takes the expression given in Table S2 which depends on the ratios $[\langle k\rangle/f_{\mathrm{diff}}]^{\mathrm{Edge,Max}}$ and $[\langle k\rangle/f_{\mathrm{diff}}]^{\mathrm{SDS}}$ and the fraction $f_{\mathrm{Edge}}^{\mathrm{Max}}$, which are defined in the main text.

The $[\langle k\rangle/f_{\mathrm{diff}}]^{\mathrm{Mid}}$ ratio representing the PyCX-PAMAM-G0 samples located in the hydrophobic middle region of the NSSA that would not undergo a conformational inversion due to its low HG was obtained by extrapolating the $[\langle k\rangle/f_{\mathrm{diff}}]^{\mathrm{exp}}$-*vs.*-$f_{\mathrm{Mid}}$ trends for the PyCX-PAMAM-G0 samples with $X = 4$, 6, and 8 in Fig. 3A in the main text to $f_{\mathrm{Edge}} = 0$ representing a hypothetical infinitely long cylinder to yield their $[\langle k\rangle/f_{\mathrm{diff}}]^{\mathrm{exp}}(f_{\mathrm{Edge}} = 0)$ value. The $[\langle k\rangle/f_{\mathrm{diff}}]^{\mathrm{exp}}(f_{\mathrm{Edge}} = 0)$ values of the PyCX-PAMAM-G0 dendrimers with $X = 4$, 6, and 8 were plotted as a function of $[Py]_{\mathrm{loc}}$ in Fig. S1, where $[Py]_{\mathrm{loc}}$ is the local pyrene concentration of the dendrimer, which was calculated earlier by assuming that their internal segments obey Gaussian statistics (*45*). The $[\langle k\rangle/f_{\mathrm{diff}}]^{\mathrm{exp}}(f_{\mathrm{Edge}} = 0)$ ratios of the PyCX-PAMAM-G0 samples with the shorter linkers aligned around a straight line passing through the origin as expected since the internal segments of these dendrimers obeyed Gaussian statistics, which could be further used as a calibration curve to estimate the $[\langle k\rangle/f_{\mathrm{diff}}]^{\mathrm{exp}}(f_{\mathrm{Edge}} = 0)$ ratios taken as the $[\langle k\rangle/f_{\mathrm{diff}}]^{\mathrm{Mid}}$ ratio of the PyC10- and PyC12-PAMAM-G0 samples, whose values have been reported in Table S1.

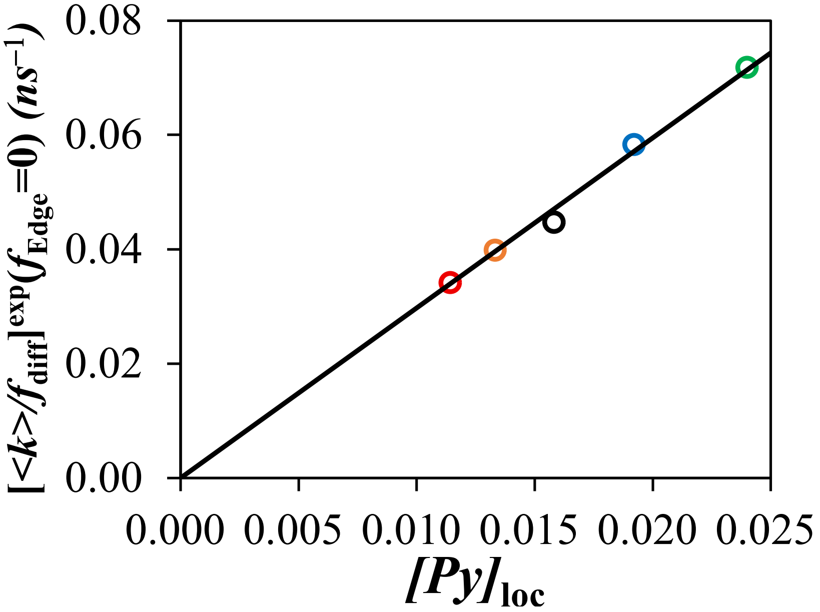

Fig. S1. Plot of $[\langle k\rangle/f_{\mathrm{diff}}]^{\mathrm{exp}}(f_{\mathrm{Edge}}=0)$ as a function of $[Py]_{\mathrm{loc}}$. Color symbols: (green) PyC4-PAMAM-G0, (blue) PyC6-PAMAM-G0, (black) PyC8-PAMAM-G0, (orange) PyC10-PAMAM-G0, and (red) PyC12-PAMAM-G0. [SDS] = 50 mM, [Py] = 2.4 μM.

The constant $[\langle k\rangle/f_{\mathrm{diff}}]^{\mathrm{Edge,Max}}$ ratio obtained at NaCl concentrations higher than ~200 mM, was determined by entering $f_{\mathrm{Edge}}^{\mathrm{Max}}$ at the intercept of the two straight lines in Fig. 3A into Eq. 1 and equating $[\langle k\rangle/f_{\mathrm{diff}}]^{\mathrm{th}}$ to $[\langle k\rangle/f_{\mathrm{diff}}]^{\mathrm{exp}}$. The $[\langle k\rangle/f_{\mathrm{diff}}]^{\mathrm{Edge,Max}}$ ratios retrieved for the PyC10- and PyC12-PAMAM-G0 samples are listed in Table S1 along with their $f_{\mathrm{Edge}}^{\mathrm{Max}}$ and

[$<k>/f_{diff}$]^{SDS} values. [$<k>/f_{diff}$]^{Edge,Max} is close to 10-times larger than [$<k>/f_{diff}$]^{Mid} reflecting the much higher HG^{Edge,Max} and degree of conformational inversion experienced by the dendrimers with the longer decanoyl and dodecanoyl linkers in the polar edge region of the NSSA compared to the apolar middle region, where HG^{Mid} ~ 0. For NaCl concentrations lower than 200 mM where $f_{Edge}^{Max} \leq f_{Edge} \leq 1.0$, the decrease in [$<k>/f_{diff}$]^{exp} with decreasing f_{Edge}, due to the partitioning of the dendrimers between the edges with a large [$<k>/f_{diff}$]^{Edge} and the Middle region with a small [$<k>/f_{diff}$]^{Mid}, is offset by the large increase experienced by [$<k>/f_{diff}$]^{Edge} from [$<k>/f_{diff}$]^{SDS} to [$<k>/f_{diff}$]^{Edge,Max} in Fig. 3B. The outcome is the linear decrease in [$<k>/f_{diff}$]^{exp} with decreasing f_{Edge} observed in Fig. 3A for the dendrimers with the longer linkers.

Using the [$<k>/f_{diff}$]^{Mid}, [$<k>/f_{diff}$]^{Edge,Max}, and [$<k>/f_{diff}$]^{SDS} ratios and f_{Edge}^{Max} listed in Table S1, the theoretical [$<k>/f_{diff}$]th ratio could be calculated with Eq. 1 using the expression of [$<k>/f_{diff}$]^{Edge} in Table S2. The excellent agreement found between the [$<k>/f_{diff}$]th and [$<k>/f_{diff}$]^{exp} ratios in Fig. 3A validates the parametrization of the experimental data assuming the existence of two distinct regions in NSSA, namely the Edge and Middle regions.

Equations used to determine the average end-to-end distance of the dendrimers

Eq. S1 derived earlier (*45*) was used to calculate the squared average end-to-end distance of the PyCX-PAMAM-G0 dendrimers with *a* (= *X*), *b* (= 7), and *c* (= 4) representing the number of carbon atoms in the linker connecting the pyrene moiety to the PAMAM-G0 dendrimer, the number of non-hydrogen atom in the aminoethyl propionamide building block of the PAMAM dendrimer, and the diaminoethylene core of the dendrimer, respectively.

$$L_{Py}^2(N) = \left(2a + \frac{\left[2-2^{N+2}+(N+1)\times 2^{N+3}\right]b + 2^{N+1}c + 2^{N+1} - 1}{2^{N+2}-1} \right) \times l^2 \qquad \text{(S1)}$$

Derivation used for the Spatial Partitioning Theory

The dendrimers (D) are assumed to partition themselves between the Edge and Middle Regions with a partition coefficient (*K*) expressed in Eq. S2. In Eq. S2, $[D]_{Edge}$ and $[D]_{Mid}$ are the dendrimer concentrations in the Edge and Middle Regions, respectively.

$$K = \frac{[D]_{Edge}}{[D]_{Mid}} \qquad \text{(S2)}$$

A relationship exists between the total concentration ([*D*]) of the dendrimers in the micellar aggregates and $[D]_{Mid}$ and $[D]_{Edge}$, as described in Eq. S3, where V_{Mid} and V_{Edge} are the volumes of the Middle and Edge Regions, respectively.

$$[D]_{Mid}\times V_{Mid} + [D]_{Edge}\times V_{Edge} = [D]\times(V_{Mid} + V_{Edge}) \qquad \text{(S3)}$$

Noting that the volume fractions f_{Mid} and f_{Edge} are simply equal to $V_{Mid}/(V_{Mid}+V_{Edge})$ and $V_{Edge}/(V_{mid}+V_{Edge})$, respectively, Eq. S3 can be rewritten into Eq. S4.

$$[D]_{Mid}\times f_{Mid} + [D]_{Edge}\times f_{Edge} = [D] \tag{S4}$$

Combining Eqs. S2 and S4 yields an expression for $[D]_{Mid}$ and $[D]_{Edge}$ given in Eqs. S5 and S6, respectively.

$$[D]_{Mid} = \frac{[D]}{f_{Mid} + K\times f_{Edge}} \tag{S5}$$

$$[D]_{Edge} = \frac{K\times[D]}{f_{Mid} + K\times f_{Edge}} \tag{S6}$$

The experimental quantity $[<k>/f_{diff}]^{exp}$, used to represent the local pyrene concentration ($[Py]_{loc}$) in the PyCX-PAMAM-G0 dendrimers embedded in the micellar aggregates, is thus represented by the quantity $[<k>/f_{diff}]^{th}$ being the average between those dendrimers located in the Middle and Edge Regions as illustrated in Eq. S7.

$$[D]_{Mid}V_{Mid}\times[<k>/f_{diff}]^{Mid} + [D]_{Edge}V_{Edge}\times[<k>/f_{diff}]^{Edge} = [D]\times(V_{Edge} + V_{Mid})\times[<k>/f_{diff}]^{th} \tag{S7}$$

Taking advantage of the definition of f_{Mid} and f_{Edge}, Eq. S7 can be rewritten into Eq. S8.

$$[<k>/f_{diff}]^{th} = f_{Mid}[<k>/f_{diff}]^{Mid}\times\frac{[D]_{Mid}}{[D]} + f_{Edge}[<k>/f_{diff}]^{Edge}\times\frac{[D]_{Edge}}{[D]} \tag{S8}$$

Finally, Eqs. S5 and S6 can be used to replace the ratios $[D]_{Mid}/[D]$ and $[D]_{Edge}/[D]$ in Eq. S8 to yield Eq. S9.

$$[<k>/f_{diff}]^{th} = [<k>/f_{diff}]^{Mid}\times\frac{f_{Mid}}{f_{Mid}+K\times f_{Edge}} + [<k>/f_{diff}]^{Edge}\times\frac{K\times f_{Edge}}{f_{Mid}+K\times f_{Edge}} \tag{S9}$$

Using the parameters provided in Table S1 for PyC10-PAMAM-G0, Eq. S9 can be used to assess the trends obtained for $[<k>/f_{diff}]^{th}$ when K equals 0.2 (dendrimers bind preferentially to the Middle Region), 1 (no preference, assumption in the manuscript), and 5 (dendrimers bind preferentially to the Edge Region). The resulting plots for $[<k>/f_{diff}]^{th}$ as a function of f_{Edge} for f_{Edge} values lower than f_{Edge}^{Max} are shown in Figure S2.

According to the plots shown in Figure S2, the only case where $[<k>/f_{diff}]^{th}$ yields a straight line is for $K = 1$, i.e. no preferential partitioning, in which case Eq. S9 simplifies into Eq. S10, which is equivalent to Eq. 2 used in the main text to represent the experimental results in Figure 3A.

$$[<k>/f_{\mathrm{diff}}]^{\mathrm{th}} = [<k>/f_{\mathrm{diff}}]^{\mathrm{Mid}} \times f_{\mathrm{Mid}} + [<k>/f_{\mathrm{diff}}]^{\mathrm{Edge}} \times f_{\mathrm{Edge}} \quad \text{(S10)}$$

Based on the plot shown in Figure S2, it would seem that the dendrimers do not partition preferentially in either the Edge or the Middle Regions since the best agreement with the experimental $[<k>/f_{\mathrm{diff}}]^{\mathrm{exp}}$ trends is obtained assuming $K = 1$. Eqs. S10 and 2 are the basis for the Spatial Partitioning Theory describing the distribution of the PyCX-PAMAM-G0 dendrimers between the Middle and Edge Regions.

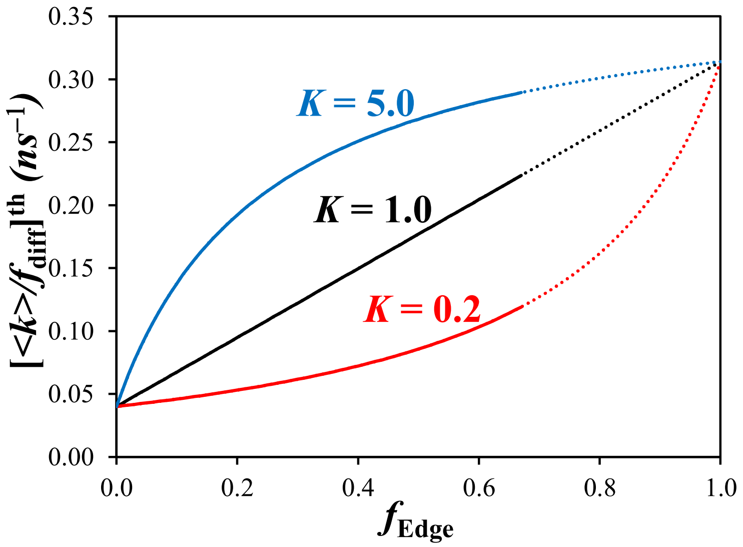

Figure S2. Plot of $[<k>/f_{\mathrm{diff}}]^{\mathrm{th}}$ as a function of f_{Edge} for a partition coefficient (K) equal to (blue) 5, (black) 1, and (red) 0.2 starting from $f_{\mathrm{Edge}}^{\mathrm{Max}} = 0.67$ for PyC10-PAMAM-G0.

Parametrization of the N_{agg}([NaCl]) trends

To calculate the volume fraction f_{Edge} in Eq. 2, the N_{agg}([NaCl]) values of NSSA for SDS aqueous solutions with NaCl reported in the literature (*4*,*9*) were plotted as a function of NaCl concentration in Fig. S3 and fitted with the empirical Eq. S11.

$$N_{agg}([\mathrm{NaCl}]) = (46.761\times[\mathrm{NaCl}]^4-5.803\times[\mathrm{NaCl}]^3+2.051\times[\mathrm{NaCl}]^2+0.4369\times[\mathrm{NaCl}]+0.979)\times (146.17\times[\mathrm{NaCl}] + 74.643) \quad \text{(S11)}$$

The N_{agg}([NaCl]) values calculated with Eq. S11 were used to determine f_{Edge} with Eq. 2.

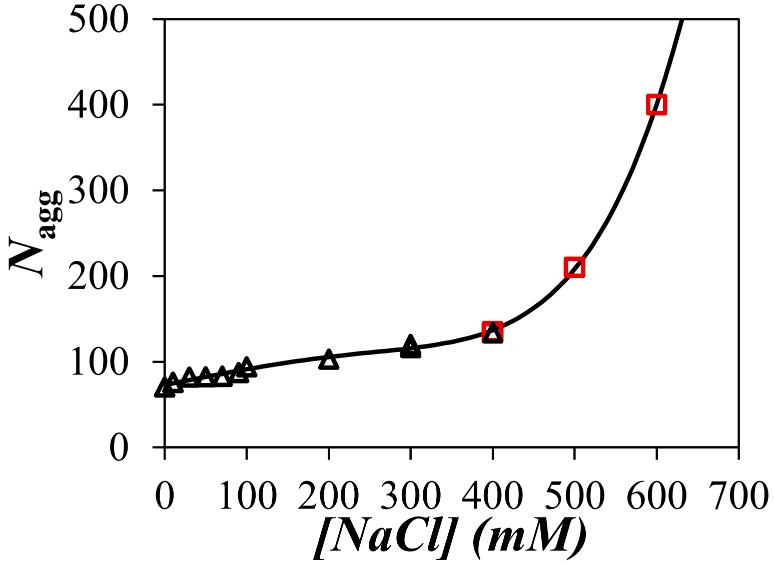

Figure S3. Plot of N_{agg} as a function of [NaCl] for N_{agg} values obtained from references (black triangles (*62*)) and (red squares (*47*))

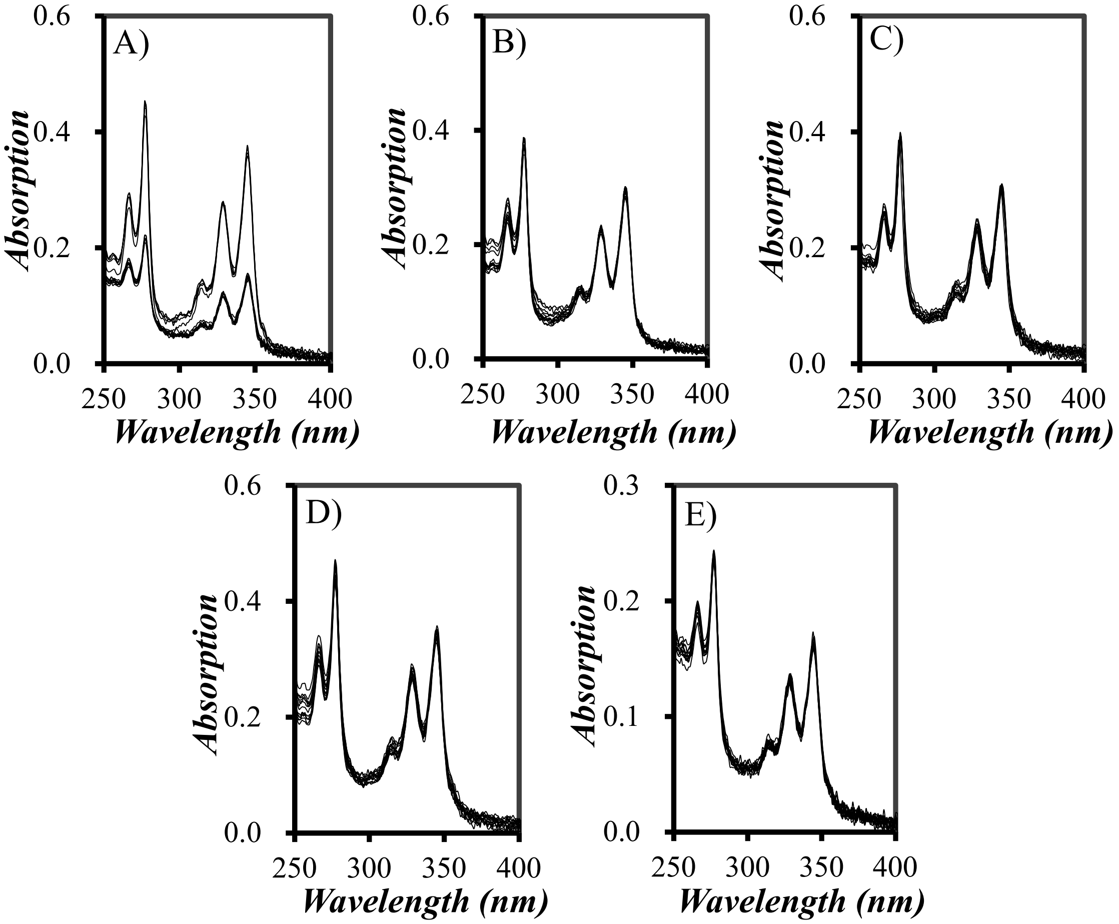

Figure S4. Baseline corrected UV-Vis absorption spectra of A) PyC4-PAMAM-G0, B) PyC6-PAMAM-G0, C) PyC8-PAMAM-G0, D) PyC10-PAMAM-G0, and E) PyC12-PAMAM-G0 in 50 mM SDS aqueous solution with [NaCl] ranging from 0 to 620 mM. No significant absorption is observed at longer wavelength. The difference in absorption observed in Figure 1A is because the UV-vis experiment is conducted separately under 0-100mM and 200mM-620 mM range of [NaCl] with different PyC4-PAMAM-G0 concentrations. The different concentrations used in these experiments had no affect on their conformation and the fluorescence results.

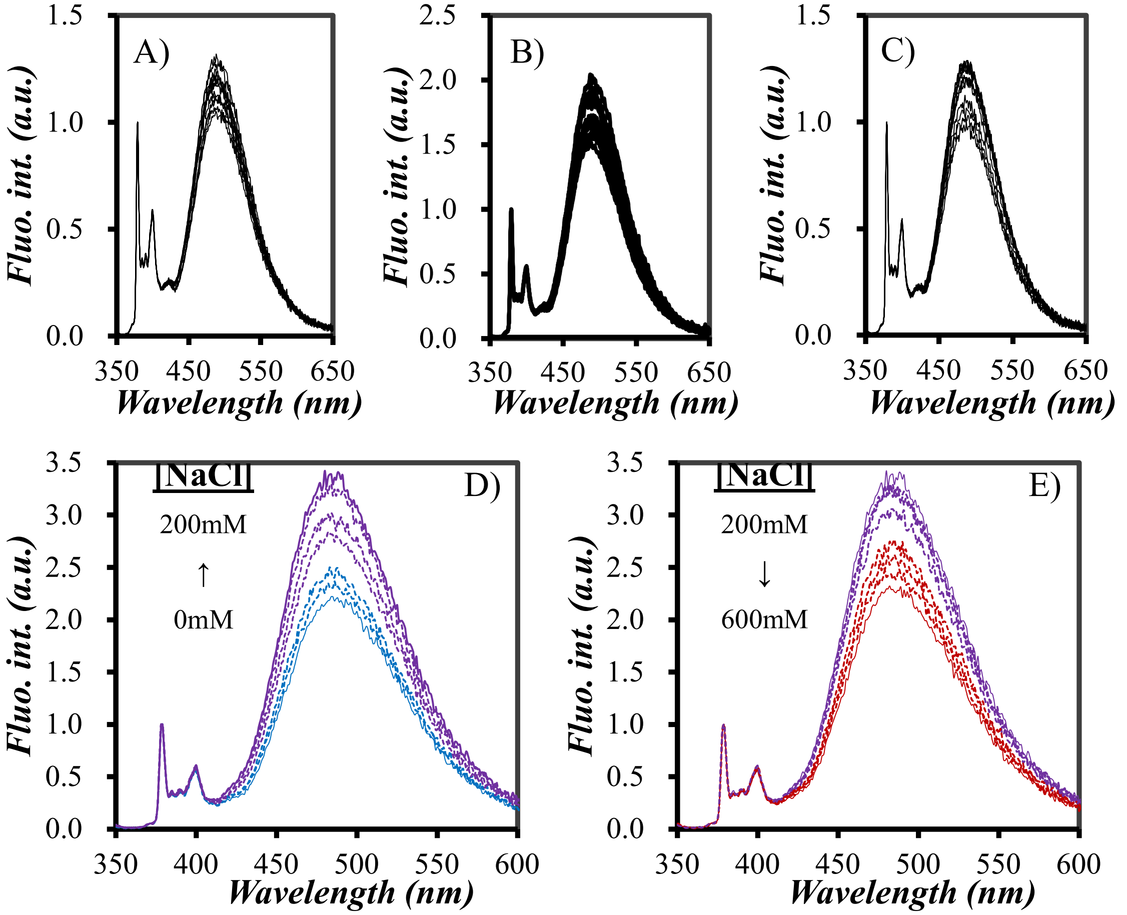

Figure S5. Fluorescence spectra of A) PyC4-PAMAM-G0, B) PyC6-PAMAM-G0, and C) PyC8-PAMAM-G0 in 50 mM SDS aqueous solution with [NaCl] ranging from 0 to 620 mM showing a small variation with ionic strength, and fluorescence spectra of PyC10-PAMAM-G0 in 50 mM SDS aqueous solution with [NaCl] ranging from D) 0 to 200 mM showing a significant increase pyrene excimer fluorescence, and E) from 200 to 620 mM exhibiting an equivalent decrease in pyrene excimer fluorescence. The trends in Figures S2D and E resemble the ones obtained for PyC12-PAMAM-G0 presented in the main text and these variations in pyrene excimer fluorescence were due to a similar conformational inversion.

Table S1. $[<k>/f_{diff}]^{Mid}$, $[<k>/f_{diff}]^{Edge,Max}$, and $[<k>/f_{diff}]^{SDS}$ ratios and f_{Edge}^{Max} determined experimentally and used in Eq. 1 to predict the theoretical $[<k>/f_{diff}]^{th}$ ratio.

	$[<k>/f_{diff}]^{Mid}$	$[<k>/f_{diff}]^{Edge,Max}$	$[<k>/f_{diff}]^{SDS}$	f_{Edge}^{Max}
PyC10-PAMAM-G0	0.040	0.314	0.109	0.67
PyC12-PAMAM-G0	0.034	0.351	0.134	0.73

Table S2. Mathematical expression for the function $[<k>/f_{diff}]^{Edge}(f_{Edge})$ in Eq. 1

$0 < f_{Edge} < f_{Edge}^{Max}$	$f_{Edge}^{Max} < f_{Edge} < 1.0$
$[<k>/f_{diff}]^{Edge,Max}$	$\dfrac{[<k>/f_{diff}]^{Edge,Max} f_{Edge}^{Max}(1-f_{Edge}) + [<k>/f_{diff}]^{SDS}(f_{Edge}-f_{Edge}^{Max})}{f_{Edge}(1-f_{Edge}^{Max})}$

Table S3. Parameters retrieved from the MFA of the monomer fluorescence decays of the PyCX-PAMAM-G0 dendrimers in 50 mM SDS aqueous at various NaCl concentrations.

**Linker**	**[NaCl] *(mM)***	a_1	τ_1 (ns)	a_2	τ_2 (ns)	a_3	τ_3 (ns)	f_{Mfree}	τ_M (ns)	χ^2
**C4**	0	0.34	5.33	0.50	11.68	0.06	32.05	0.102	132	1.06
	11	0.28	4.56	0.56	10.96	0.06	33.17	0.101		1.20
	20	0.36	5.20	0.45	11.91	0.09	25.29	0.103		1.13
	49	0.32	4.94	0.51	11.42	0.07	31.92	0.101		1.08
	93	0.29	5.19	0.48	11.56	0.12	25.53	0.108		1.17
	193	0.39	5.51	0.47	12.85	0.11	26.72	0.036		1.16
	284	0.34	5.60	0.49	13.39	0.13	26.60	0.040		1.19
	373	0.41	6.09	0.38	13.63	0.16	24.89	0.039		1.05
	458	0.25	5.03	0.50	12.61	0.20	25.16	0.042		1.20
	541	0.35	5.40	0.52	14.81	0.09	30.38	0.040		1.07
	566	0.32	5.31	0.51	14.63	0.14	28.18	0.041		1.22
	592	0.34	5.40	0.51	14.74	0.11	29.20	0.040		1.11
	618	0.31	5.68	0.50	14.74	0.14	28.01	0.044		1.10

Linker	[NaCl] *(mM)*	a_1	τ_1 *(ns)*	a_2	τ_2 *(ns)*	a_3	τ_3 *(ns)*	f_{Mfree}	τ_M *(ns)*	χ^2
C6	0	0.19	4.50	0.69	12.21	0.11	26.91	0.014	127	1.08
	10	0.23	5.27	0.65	12.45	0.11	26.97	0.014		1.10
	20	0.34	5.32	0.62	13.24	0.03	39.06	0.013		1.07
	48	0.27	5.31	0.65	13.66	0.07	32.97	0.017		1.15
	92	0.34	5.52	0.60	13.65	0.04	40.15	0.015		1.19
	198	0.18	3.89	0.72	12.73	0.09	31.56	0.011		1.15
	289	0.16	3.70	0.63	11.55	0.20	23.13	0.014		1.18
	376	0.19	3.79	0.71	12.31	0.09	29.23	0.013		1.19
	460	0.20	4.05	0.64	12.58	0.16	27.21	0.014		1.00
	542	0.26	5.44	0.60	13.96	0.13	27.92	0.015		1.10
	567	0.21	4.80	0.62	13.95	0.16	28.75	0.015		1.16
	593	0.25	5.59	0.56	14.14	0.17	26.30	0.016		1.27
	617	0.16	3.73	0.69	14.80	0.13	33.27	0.015		1.16

Linker	[NaCl] *(mM)*	a_1	τ_1 *(ns)*	a_2	τ_2 *(ns)*	a_3	τ_3 *(ns)*	f_{Mfree}	τ_M *(ns)*	χ^2
C8	0	0.26	5.367	0.57	15.26	0.13	33.33	0.036	127	1.19
	10	0.19	4.13	0.62	13.83	0.16	33.09	0.033		1.11
	19	0.19	3.657	0.65	13.03	0.13	32.18	0.034		1.18
	47	0.21	4.69	0.60	14.15	0.15	33.64	0.036		1.12
	92	0.26	3.86	0.56	13.36	0.14	30.85	0.036		1.13
	198	0.29	4.08	0.54	14.89	0.14	33.44	0.037		1.15
	288	0.21	3.10	0.53	12.24	0.22	29.42	0.040		1.07
	382	0.33	3.64	0.53	15.00	0.10	36.33	0.037		1.15
	461	0.23	2.86	0.50	12.47	0.23	30.33	0.041		1.14
	542	0.30	3.60	0.49	15.25	0.18	33.26	0.040		1.15
	570	0.25	3.79	0.57	16.21	0.14	39.97	0.038		1.20
	595	0.28	3.59	0.51	15.68	0.17	33.52	0.040		1.21
	621	0.24	4.13	0.55	16.74	0.17	37.77	0.041		1.20

Linker	[NaCl] *(mM)*	a_1	τ_1 (ns)	a_2	τ_2 (ns)	a_3	τ_3 (ns)	f_{Mfree}	τ_M (ns)	χ^2
**C10**	0	0.28	2.55	0.55	11.43	0.14	26.54	0.027	132	1.13
	11	0.36	2.51	0.54	11.27	0.07	31.55	0.025		1.14
	20	0.34	1.83	0.52	10.25	0.11	26.86	0.026		1.11
	48	0.39	2.08	0.49	9.96	0.09	26.78	0.028		1.19
	93	0.39	1.74	0.46	9.00	0.12	23.96	0.030		1.12
	193	0.38	1.39	0.34	6.28	0.20	15.52	0.072		1.01
	286	0.38	1.32	0.31	6.02	0.24	15.33	0.070		1.05
	375	0.37	1.86	0.34	6.78	0.22	17.05	0.076		1.18
	460	0.37	1.79	0.31	7.73	0.25	17.96	0.075		1.19
	542	0.36	2.29	0.35	10.07	0.22	20.84	0.075		1.15
	566	0.31	1.93	0.41	10.20	0.25	25.39	0.026		1.19
	591	0.32	2.35	0.44	11.55	0.22	27.14	0.027		1.15
	622	0.28	2.20	0.26	8.83	0.39	21.61	0.071		1.08

Linker	[NaCl] *(mM)*	a_1	τ_1 (ns)	a_2	τ_2 (ns)	a_3	τ_3 (ns)	f_{Mfree}	τ_M (ns)	χ^2
**C12**	0	0.32	2.98	0.49	11.95	0.12	35.32	0.065	127	1.19
	10	0.34	2.25	0.48	10.57	0.11	34.29	0.065		1.19
	20	0.36	1.67	0.44	9.34	0.13	30.30	0.069		1.05
	48	0.41	1.90	0.42	9.99	0.10	35.35	0.071		1.11
	94	0.45	2.28	0.38	10.72	0.09	34.52	0.080		1.07
	193	0.40	1.59	0.40	8.24	0.12	29.48	0.076		1.11
	284	0.44	2.16	0.39	9.62	0.09	33.86	0.078		1.11
	372	0.40	1.98	0.38	8.44	0.14	26.81	0.075		1.15
	459	0.40	2.16	0.41	9.82	0.11	32.39	0.070		1.10
	537	0.38	2.23	0.41	10.76	0.14	32.01	0.066		1.13
	562	0.34	2.31	0.42	9.90	0.17	30.70	0.064		1.13
	589	0.31	2.04	0.41	10.18	0.22	29.25	0.064		1.18
	615	0.35	2.65	0.40	11.86	0.18	31.26	0.062		1.13

Table S4. Parameters retrieved from the MFA of the excimer fluorescence decays of PyCX-PAMAM-G0 dendrimers in 50 mM SDS aqueous solution at various NaCl concentrations.

Linker	[NaCl] (mM)	$f_{EdiffE0}$	f_{EdiffD}	τ_{E0} (ns)	τ_D (ns)	τ_S (ns)	f_{EE0}	f_{ED}	f_{ES}^*	χ^2
	0	0.26	0.39	40.05	54.22		0.00	0.09	0.26	1.06
	11	0.33	0.33	43.21	55.40		0.00	0.04	0.29	1.20
	20	0.31	0.36	37.87	55.30		0.00	0.06	0.27	1.13
	49	0.30	0.38	44.70	53.57		0.00	0.01	0.31	1.08
	93	0.31	0.36	38.74	55.34		0.00	0.06	0.26	1.17
	193	0.26	0.34	44.90	56.20		0.00	0.01	0.39	1.16
**C4**	284	0.26	0.33	40.15	57.57	3.5	0.00	0.03	0.04	1.19
	373	0.26	0.33	43.65	56.87		0.00	0.01	0.40	1.05
	458	0.24	0.36	39.40	56.77		0.00	0.04	0.36	1.20
	541	0.33	0.27	57.02	44.14		0.01	0.00	0.38	1.07
	566	0.25	0.35	42.36	55.32		0.00	0.04	0.36	1.22
	592	0.25	0.36	42.26	56.37		0.00	0.01	0.38	1.11
	618	0.26	0.34	38.37	57.37		0.00	0.04	0.36	1.10

Linker	[NaCl] (mM)	$f_{EdiffE0}$	f_{EdiffD}	τ_{E0} (ns)	τ_D (ns)	τ_S (ns)	f_{EE0}	f_{ED}	f_{ES}^*	χ^2
	0	0.49	0.40	40.77	57.38		0.00	0.06	0.05	1.08
	10	0.23	0.64	35.00	52.01		0.00	0.12	0.00	1.10
	20	0.53	0.35	50.30	50.28		0.00	0.02	0.10	1.07
	48	0.47	0.41	41.10	55.88		0.01	0.08	0.04	1.15
	92	0.38	0.49	50.76	50.48		0.00	0.02	0.11	1.19
	198	0.32	0.58	43.45	52.18		0.00	0.06	0.04	1.15
**C4**	289	0.55	0.36	41.32	57.42	3.5	0.01	0.08	0.00	1.18
	376	0.39	0.52	47.38	52.06		0.00	0.06	0.04	1.19
	460	0.39	0.52	39.35	53.74		0.00	0.08	0.01	1.00
	542	0.41	0.49	50.20	50.12		0.01	0.06	0.04	1.10
	567	0.39	0.51	43.54	52.78		0.02	0.08	0.00	1.16
	593	0.36	0.54	49.77	51.22		0.00	0.06	0.04	1.27
	617	0.40	0.51	53.98	45.37		0.01	0.07	0.00	1.16

Linker	[NaCl] *(mM)*	$f_{EdiffE0}$	f_{EdiffD}	τ_{E0} (ns)	τ_D (ns)	τ_S (ns)	f_{EE0}	f_{ED}	f_{ES}^*	χ^2
C8	0	0.40	0.50	49.41	49.61	3.5	0.00	0.06	0.08	1.19
	10	0.56	0.32	51.79	42.88		0.00	0.07	0.05	1.11
	19	0.34	0.54	47.52	51.71		0.02	0.02	0.09	1.18
	47	0.39	0.47	52.10	46.15		0.05	0.07	0.01	1.12
	92	0.37	0.50	52.21	49.93		0.00	0.01	0.12	1.13
	198	0.36	0.48	50.62	50.72		0.00	0.06	0.10	1.15
	288	0.35	0.49	47.41	51.39		0.11	0.03	0.03	1.07
	382	0.37	0.47	51.08	51.07		0.00	0.04	0.12	1.15
	461	0.37	0.47	51.10	49.45		0.01	0.08	0.07	1.14
	542	0.34	0.51	50.86	50.91		0.00	0.02	0.13	1.15
	570	0.34	0.52	49.24	51.09		0.00	0.09	0.05	1.20
	595	0.30	0.56	50.10	52.16		0.00	0.01	0.13	1.21
	621	0.35	0.50	50.54	50.91		0.00	0.08	0.07	1.20

Linker	[NaCl] *(mM)*	$f_{EdiffE0}$	f_{EdiffD}	τ_{E0} (ns)	τ_D (ns)	τ_S (ns)	f_{EE0}	f_{ED}	f_{ES}^*	χ^2
C10	0	0.34	0.42	42.56	55.56	3.5	0.06	0.15	0.03	1.13
	11	0.17	0.57	43.24	53.50		0.01	0.19	0.06	1.14
	20	0.11	0.65	32.82	52.04		0.00	0.23	0.02	1.11
	48	0.27	0.45	43.50	55.44		0.08	0.14	0.07	1.19
	93	0.08	0.63	33.91	52.69		0.08	0.18	0.03	1.12
	193	0.05	0.64	48.82	51.35		0.06	0.25	0.00	1.01
	286	0.07	0.62	34.59	51.65		0.05	0.27	0.00	1.05
	375	0.11	0.54	46.08	52.24		0.10	0.25	0.00	1.18
	460	0.07	0.66	16.27	50.89		0.00	0.26	0.01	1.19
	542	0.21	0.51	50.75	51.63		0.09	0.19	0.00	1.15
	566	0.24	0.51	45.49	52.22		0.11	0.14	0.00	1.11
	591	0.31	0.54	49.64	49.70		0.02	0.10	0.03	1.05
	622	0.23	0.57	36.68	53.59		0.00	0.19	0.00	1.08

Linker	[NaCl] *(mM)*	$f_{EdiffE0}$	f_{EdiffD}	τ_{E0} (ns)	τ_D (ns)	τ_S (ns)	f_{EE0}	f_{ED}	f_{ES}^{*}	χ^2
**C12**	0	0.16	0.44	50.11	50.20	3.5	0.19	0.19	0.02	1.12
	10	0.12	0.45	51.18	51.07		0.22	0.17	0.04	1.13
	20	0.13	0.45	44.38	52.68		0.18	0.19	0.04	1.10
	48	0.09	0.43	50.77	50.39		0.22	0.21	0.05	1.14
	94	0.00	0.45	43.42	51.66		0.23	0.29	0.02	1.20
	193	0.02	0.42	46.67	51.45		0.30	0.26	0.00	1.11
	284	0.03	0.43	50.53	50.41		0.27	0.26	0.00	1.11
	372	0.07	0.45	49.63	49.60		0.25	0.21	0.02	1.15
	459	0.41	0.17	50.64	50.51		0.23	0.17	0.02	1.10
	537	0.20	0.44	50.06	50.01		0.18	0.16	0.02	1.13
	562	0.28	0.40	50.48	50.44		0.14	0.11	0.07	1.13
	589	0.48	0.21	49.43	51.42		0.23	0.05	0.02	1.18
	615	0.32	0.40	50.43	50.38		0.10	0.08	0.09	1.13

Listing of the program used to conduct the global MFA of the fluorescence decays

```
c   Program created on January 19th, 2010


c   This program fits the monomer and excimer decays simultaneously
c   with three exponentials
c   The monomer decay is fitted with three exponential + one for
c   unquenched monomers.
c   The excimer decay is fitted with the same three exponentials + one
c   for excited ground-state dimers (E0*) and one for
c   long lived dimers (D*).
c   The same excimer formation rate constantis give E0* and D*.


    implicit double precision (a-h,o-z)

    parameter (mfit=17,ma=17,nca=17)

    dimension y1(5000),lista(mfit),a(mfit),
     & covar(mfit,mfit),alpha(mfit,mfit),
     & e1(5000),da(mfit),beta(mfit)
     & ,dyda(mfit),ym(5000),res1(5000),auto1(5000)
     & ,e2(5000),y2(5000),res2(5000),auto2(5000)
     & ,nstart1(3),nstart2(3),f(5000)

    real*8 taum,chisq,tpc1,tpc2


10  format(A)
    character *30 DD1,DD2

    write (*,*) 'How many chanels do you want to work with
     & for the monomer decay?'
    read (*,*) ndata1

c   write (*,*) 'What is the name of your lamp file ?'
c   read(*,10) DD

c   open (1,file=DD,status='old')

c   read(1,*) (e(i),i=1,ndata)

c   close(1)
```

```
c   write (*,*) 'what is the name of your fluorescence decay?'
c   read(*,10) DD

c   open(1,file=DD,status='old')

c   read(1,*) (y(i),i=1,ndata)

c   close(1)

c   write (*,*) 'What is your file''s name for the monomer?'
c   read(*,10) DD1

c   open(1,file=DD1,status='old')

c   do 15 i=1,ndata1

c   read(1,*) e1(i),y1(i)

c15 continue

c   close (1)

    write (*,*) 'What is your lamp''s filename for
     & the monomer decay?'
    read(*,10) DD1

    open(1,file=DD1,status='old')

    do 13 i=1,10
13  read(1,*)

    do 15 i=1,ndata1

    read(1,*) x,e1(i)

15  continue

    close (1)


    write (*,*) 'What is your monomer decay''s filename?'
    read(*,10) DD1
```

```
      open(1,file=DD1,status='old')

      do 14 i=1,10
14    read(1,*)

      do 16 i=1,ndata1

      read(1,*) x,y1(i)

16    continue

      close (1)

      write (*,*) 'How many chanels do you want to work with
     & for the excimer decay?'
      read (*,*) ndata2


c     write (*,*) 'What is your file''s name for the excimer?'
c     read(*,10) DD2

c     open(1,file=DD2,status='old')

c     do 16 i=1,ndata2

c     read(1,*) e2(i),y2(i)

c16   continue

c     close (1)


      write (*,*) 'What is your lamp''s filename for
     & the excimer decay?'
      read(*,10) DD2

      open(1,file=DD2,status='old')

      do 313 i=1,10
313   read(1,*)

      do 315 i=1,ndata2

      read(1,*) x,e2(i)
```

```
315 continue

    close (1)


    write (*,*) 'What is your excimer decay''s filename?'
    read(*,10) DD2

    open(1,file=DD2,status='old')

    do 314 i=1,10
314 read(1,*)

    do 316 i=1,ndata2

    read(1,*) x,y2(i)

316 continue

    close (1)

    write (*,*) 'What is the lifetime of the reference compound
     & for the monomer decays?'
    read(*,*) taur1

    write (*,*) 'What is your time per chanel for the monomer decays?'
    read(*,*) tpc1

    taur1 = exp(-tpc1/taur1)

    alold2 = e1(1)

    do 40 i=2,ndata1

    alold1 = e1(i)
    e1(i) = e1(i) - alold2*taur1
    alold2 = alold1

    if(e1(i).lt.0) e1(i)=0.0001

40  continue
```

```
      write (*,*) 'What is the lifetime of the reference compound
     & for the excimer decays?'
      read(*,*) taur2

      write (*,*) 'What is your time per chanel for the excimer decays?'
      read(*,*) tpc2

      taur2 = exp(-tpc2/taur2)

      alold2 = e2(1)

      do 41 i=2,ndata2

      alold1 = e2(i)
      e2(i) = e2(i) - alold2*taur2
      alold2 = alold1

      if(e2(i).lt.0) e2(i)=0.0001

41    continue

      imax = 0

      do 500 i=2,ndata1

      if (e1(i).gt.emax) then
      imax = i
      emax = e1(i)
      endif

500   continue


      write (*,511)
      write (*,*) '_________________________________________________'
511   format(10H   channel,10H     lamp,10H    decay)

      do 520 k=imax-15,imax+10

      if (k.gt.1) then
      write (*,512) k,e1(k),y1(k)
512   format (3H   ,I4,3H   ,2(2H  ,F7.1,1H ))
      endif
520   continue
```

```
      write (*,*) 'From which channel do you wish to start
     &  your analysis for the monomer decay?'
      read(*,*) nstart1(3)

      write (*,*) 'At which channel does the background noise start
     & for the monomer decay?'
      read(*,*) nstart1(1)

      write (*,*) 'At which channel does the background noise end
     & for the monomer decay?'
      read(*,*) nstart1(2)

c     sume = 0.0
c     sumy = 0.0

c     anback = nstart1(2)-nstart1(1)+1

c     do 860 i=nstart1(1),nstart1(2)

c     sume = sume + e1(i)
c     sumy = sumy + y1(i)

c860   continue

c     sume = sume/anback
c     sumy = sumy/anback

c     do 870 i=1,ndata1

c     e1(i) = e1(i) - sume
c     y1(i) = y1(i) - sumy

c     if(e1(i).lt.0.0) e1(i) = 0.00001
c     if(y1(i).lt.0.0) y1(i) = 0.00001

c870   continue


      imax = 0
      emax = 0.0

      do 501 i=2,ndata2
```

```
      if (e2(i).gt.emax) then
      imax = i
      emax = e2(i)
      endif

501   continue


      write (*,511)
      write (*,*) '________________________________________________'

      do 1522 k=imax-15,imax+10

      if (k.gt.1) then
      write (*,512) k,e2(k),y2(k)
      endif
1522    continue

      write (*,*) 'From which channel do you wish to start
     &  your analysis for the excimer decay?'
      read(*,*) nstart2(3)

      write (*,*) 'At which channel does the background noise start
     & for the excimer decay?'
      read(*,*) nstart2(1)

      write (*,*) 'At which channel does the background noise end
     & for the excimer decay?'
      read(*,*) nstart2(2)

c     sume = 0.0
c     sumy = 0.0

c     anback = nstart2(2)-nstart2(1) + 1

c     do 861 i=nstart2(1),nstart2(2)

c     sume = sume + e2(i)
c     sumy = sumy + y2(i)

c861     continue

c     sume = sume/anback
c     sumy = sumy/anback
```

```
c   do 871 i=1,ndata2

c   e2(i) = e2(i) - sume
c   y2(i) = y2(i) - sumy

c   if(e2(i).lt.0.0) e2(i) = 0.00001
c   if(y2(i).lt.0.0) y2(i) = 0.00001

c871   continue


    write (*,*) 'Give an estimate for the first decay time of
     & the monomer and excimer.'
    read(*,*) a(2)

    write (*,*) 'Give an estimate for its pre-exponential factor.'
    read (*,*) a(1)

    write (*,*) 'Give an estimate for the second decay time of
     & the monomer and excimer.'
    read(*,*) a(4)

    write (*,*) 'Give an estimate for its pre-exponential factor.'
    read (*,*) a(3)

    write (*,*) 'Give an estimate for the third decay time of
     & the monomer and excimer.'
    read(*,*) a(6)

    write (*,*) 'Give an estimate for its pre-exponential factor.'
    read (*,*) a(5)

    write (*,*) 'What is the monomer lifetime (tauM is fixed).'
    read (*,*) taum

    write (*,*) 'Give an estimate of the pre-exponential factor
     & for unquenched monomer.'
    read (*,*) a(7)

    write (*,*) 'Give an estimate of the lifetime of the GS dimers
     & (E0*).'
    read(*,*) a(10)
    write (*,*) 'Give an estimate of the pre-exponential factor of
```

```
      & this exponential.'
     read (*,*) a(8)
     write (*,*) 'Give an estimate of the contribution of the monomers
      & forming E0* by diffusion?'
     read (*,*) a(9)

     write (*,*) 'Give an estimate for the decay time of
      & the long-lived ground-state dimers (D*).'
     read(*,*) a(13)

     write (*,*) 'Give an estimate for its pre-exponential factor.'
     read (*,*) a(11)

     write (*,*) 'Give an estimate of the contribution of the monomers
      & forming D* by diffusion?'
     read (*,*) a(12)

     a(14) = .1
     a(15) = .1

     a(16) = 10.0
     a(17) = 10.0


     do 524 i=1,mfit
     lista(i) =i
524  continue

513  alambda = -0.1
     itera = 0
     kchi = 0

514  continue

     a(1) = sqrt(a(1))
     a(3) = sqrt(a(3))
     a(5) = sqrt(a(5))
     a(7) = sqrt(a(7))
     a(8) = sqrt(a(8))
     a(9) = sqrt(a(9))
     a(11) = sqrt(a(11))
     a(12) = sqrt(a(12))
     a(16) = sqrt(a(16))
     a(17) = sqrt(a(17))
```

```
      if (itera.ne.0) goto 540

515   write (*,521)
521   format(10Hiteration ,20H    amplitude       ,
     &20H    lifetime        ,20H    scattering      ,10H chisquare)
      write(*,*)'_______________________________________________________
     ____________'

c     write (*,*) 'just before entering mrqmin. taum = ',taum

540   call mrqmin(taum,y1,y2,ndata1,ndata2,a,ma,lista,mfit,covar,alpha,
     &  nca,chisq,alambda,nstart1,nstart2,e1,e2,itera,da,beta
     &  ,dyda,tpc1,tpc2,f)

      write (*,*)

      a(1) = a(1)*a(1)
      a(3) = a(3)*a(3)
      a(5) = a(5)*a(5)
      a(7) = a(7)*a(7)
      a(8) = a(8)*a(8)
      a(9) = a(9)*a(9)
      a(11) = a(11)*a(11)
      a(12) = a(12)*a(12)
      a(16) = a(16)*a(16)
      a(17) = a(17)*a(17)

      write (*,542) itera,a(1),a(2),a(14),
     &  chisq/(ndata1+ndata2-nstart1(3)-nstart2(3)+
     &  (nstart1(2)-nstart1(1))+(nstart2(2)-nstart2(1))-mfit)
542   format(I5,5H     ,3(3H   ,F10.3,7H       ),F10.3)

      write (*,543) a(16),a(17)
543   format(10HBackground,2(3H   ,F10.3,7H       ))


      write (*,523) a(3),a(4)
      write (*,523) a(5),a(6)
      write (*,523) a(7),taum

      write (*,525) a(8),a(9),a(10),a(15)
      write (*,522) a(11),a(12),a(13)
522   format(10H          ,3(3H   ,F10.3,7H       ))
```

```
523 format(10H          ,2(3H   ,F10.3,7H       ))
525 format(10H          ,4(3H   ,F10.3,7H       ))


c   write(*,*) 'alambda = ',alambda

    if (itera.eq.1) goto 514
    if(chicca.eq.chisq) kchi = kchi+1
    if (chicca.ne.chisq) chicca = chisq
    if(chicca.ne.chisq) kchi = 0
    if (kchi.gt.50) goto 550
    goto 514

550 continue

c550   write (*,*) 'Do you want to try new amplitudes and lifetimes
c     & or starting analysis channel?'
c   write (*,*) 'yes = 1'
c   read(*,*) nn
c   if (nn.eq.1) then

c   do 525 i=1,nexp
c       ii = nexp+i
c       write (*,*) 'what is your new ',i,'th lifetime?'
c       read(*,*) a(ii)
c       write (*,*) 'what is your new ',i,'th amplitude?'
c       read(*,*) a(i)
c525   continue

c   write (*,*) 'what is your new scattering factor correction?'
c   read(*,*) a(mfit)

c       write (*,*) 'what is your new starting analysis channel?'
c       read(*,*) nstart
c       goto 513
c   else
        write (*,*) 'this is the end, my friend!'
c   endif


    a(1) = sqrt(a(1))
    a(3) = sqrt(a(3))
    a(5) = sqrt(a(5))
    a(7) = sqrt(a(7))
```

```
      a(8) = sqrt(a(8))
      a(9) = sqrt(a(9))
      a(11) = sqrt(a(11))
      a(12) = sqrt(a(12))
      a(16) = sqrt(a(16))
      a(17) = sqrt(a(17))


      do 1000 i=1,ndata1

      call foncs1(i,a,ymod,dyda,ma,ndata1,e1,tpc1,taum,f)

      ym(i) = ymod
      res1(i) = y1(i) - ymod

      if (ymod.lt.1.0) then

         res1(i) = 0.0

      else

         res1(i) = res1(i)/sqrt(ymod)

      endif

      if(i.lt.nstart1(3)) res1(i) = 0.0

1000  continue

      do 1010 i=1,ndata1

      sum = sum + res1(i)*res1(i)

1010  continue

      n3 = ndata1 - nstart1(3) + 1
      an3 = ndata1 - nstart1(3) + 1

      do 1020 j=nstart1(3),n3-1

         do 1030 i=nstart1(3),ndata1-j

         auto1(j) = auto1(j) + res1(i)*res1(i+j)
```

```
1030      continue

   am = j
   am = an3 - am

   if(am.eq.0.0) then

   write (*,*) 'there is a problem!'

   endif

   auto1(j) = an3*auto1(j)/(am*sum)

1020   continue

   open(2,file='plot1.dat',status='old')

   do 1040 i=1,ndata1

   time = i*tpc1
   write (2,1050) time,e1(i),y1(i),ym(i),res1(i),auto1(i)

1050   format (4F10.2,2E12.3)

1040   continue

   close(2)

   a(1) = a(1)*a(1)
   a(3) = a(3)*a(3)
   a(5) = a(5)*a(5)
   a(7) = a(7)*a(7)
   a(8) = a(8)*a(8)
   a(9) = a(9)*a(9)
   a(11) = a(11)*a(11)
   a(12) = a(12)*a(12)
   a(16) = a(16)*a(16)
   a(17) = a(17)*a(17)

   sum = a(1)+a(3)+a(5)+a(7)

   open(2,file='plotres1',status='old')

   atcha = 0.0
```

```
      write(2,10) DD1
      write (2,1070) a(1)/sum,a(2)
      write (2,1070) a(3)/sum,a(4)
      write (2,1070) a(5)/sum,a(6)
      write (2,1070) a(7)/sum,taum
      write (2,1070) atcha,atcha
      write (2,1080) a(16)
      write (2,1080) chisq/(ndata1+ndata2-nstart1(3)-nstart2(3)+
     & (nstart1(2)-nstart1(1))+(nstart2(2)-nstart2(1))-mfit)
      write (2,1075) nstart1(3)

1070  format(2F10.5)
1075  format (I5)
1080  format (F10.5)

      close(2)


      a(1) = sqrt(a(1))
      a(3) = sqrt(a(3))
      a(5) = sqrt(a(5))
      a(7) = sqrt(a(7))
      a(8) = sqrt(a(8))
      a(9) = sqrt(a(9))
      a(11) = sqrt(a(11))
      a(12) = sqrt(a(12))
      a(16) = sqrt(a(16))
      a(17) = sqrt(a(17))

      do 2000 i=1,ndata2

      call foncs2(i,a,ymod,dyda,ma,ndata2,e2,tpc2,taum)

      ym(i) = ymod
      res2(i) = y2(i) - ymod

      if (ymod.lt.1.0) then

         res2(i) = 0.0

      else

         res2(i) = res2(i)/sqrt(ymod)
```

```
      endif

      if(i.lt.nstart2(3)) res2(i) = 0.0

2000  continue

      do 2010 i=1,ndata2

      sum = sum + res2(i)*res2(i)

2010  continue

      n3 = ndata2 - nstart2(3) + 1
      an3 = ndata2 - nstart2(3) + 1

      do 2020 j=nstart2(3),n3-1

         do 2030 i=nstart2(3),ndata2-j

         auto2(j) = auto2(j) + res2(i)*res2(i+j)

2030     continue

      am = j
      am = an3 - am

      if(am.eq.0.0) then

      write (*,*) 'there is a problem!'

      endif

      auto2(j) = an3*auto2(j)/(am*sum)

2020  continue

      open(2,file='plot2.dat',status='old')

      do 2040 i=1,ndata2

      time = i*tpc2
      write (2,1050) time,e2(i),y2(i),ym(i),res2(i),auto2(i)

2040  continue
```

```
      close(2)

c     f = a(1)*(1.0/a(2)-1.0/taum)/(1.0/a(2)-1.0/a(7))
c     f = f + a(3)*(1.0/a(4)-1.0/taum)/(1.0/a(4)-1.0/a(7))

c     fm = 1.0/(1.0 + (a(6)+a(11)-a(8)*f)/(a(8)*(a(1)+a(3))))
c     fe = fm*(a(6)-a(8)*f)/(a(8)*(a(1)+a(3)))
c     fd = fm*a(11)/(a(8)*(a(1)+a(3)))

      a(1) = a(1)*a(1)
      a(3) = a(3)*a(3)
      a(5) = a(5)*a(5)
      a(7) = a(7)*a(7)
      a(8) = a(8)*a(8)
      a(9) = a(9)*a(9)
      a(11) = a(11)*a(11)
      a(12) = a(12)*a(12)
      a(16) = a(16)*a(16)
      a(17) = a(17)*a(17)

      suma = a(1) + a(3) + a(5)
      sum = a(8) + a(9)*suma + a(11) + a(12)*suma

      fme = a(9)*suma/sum
      fmd = a(12)*suma/sum
      fe = a(8)/sum
      fd = a(11)/sum

      atcha = 0.0

      open(2,file='plotres2',status='old')

      atcha = 0.0
      write(2,10) 'sumegs33bg'
      write(2,10) DD2
      write (2,1070) fme,fmd
      write (2,1070) fe,a(10)
      write (2,1070) fd,a(13)
      write (2,1070) atcha,atcha
      write (2,1080) a(17)
      write (2,1080) chisq/(ndata1+ndata2-nstart1(3)-nstart2(3)+
       & (nstart1(2)-nstart1(1))+(nstart2(2)-nstart2(1))-mfit)
      write (2,1075) nstart2(3)
```

```
      close(2)

      end




      subroutine foncs1(i,a,ymod,dyda,ma,ndata1,e1,tpc1,taum,f)

      implicit double precision (a-h,o-z)

      real*8 a(ma),y1(5000),e1(5000),dyda(ma),f(5000)
      real*8 tedi1,ted1,tadi1,tad1,tidi1,tid1,amol,tpc1,ymod,ymod1,
     & ymod2,ymod3,taum

      a(1) = a(1)*a(1)
      a(3) = a(3)*a(3)
      a(5) = a(5)*a(5)
      a(7) = a(7)*a(7)
      a(8) = a(8)*a(8)
      a(9) = a(9)*a(9)
      a(11) = a(11)*a(11)
      a(12) = a(12)*a(12)
      a(16) = a(16)*a(16)
      a(17) = a(17)*a(17)

c     if (i.eq.1) then
c     write (*,*) 'We are in foncs1!'
c     write (*,*) 'taum = ',taum
c     endif

c     if (i.eq.1) then
c     write (*,*) 'in foncs1, tpc1 = ',tpc1
c     endif


      do 5 k=1,ma
      dyda(k) = 0.
5     continue
```

```
ymod1 = 0.0
ymod2 = 0.0
ymod3 = 0.0
ymod4 = 0.0

if (i.eq.1) goto 25

tad1 = dexp(-tpc1/a(2))
tadi1 = 1.0

ted1 = dexp(-tpc1/a(4))
tedi1 = 1.0

tid1 = dexp(-tpc1/a(6))
tidi1 = 1.0

tod1 = dexp(-tpc1/taum)
todi1 = 1.0

do 20 k=1,i

akk = k

if((k.eq.1).or.(k.eq.i)) then
amol = 0.5
else
amol = 1.0
endif

ymod1 = ymod1 + amol*e1(i-k+1)*tpc1*a(1)*tadi1
ymod2 = ymod2 + amol*e1(i-k+1)*tpc1*a(3)*tedi1
ymod3 = ymod3 + amol*e1(i-k+1)*tpc1*a(5)*tidi1
ymod4 = ymod4 + amol*e1(i-k+1)*tpc1*a(7)*todi1

dyda(2) = dyda(2) + amol*e1(i-k+1)*tpc1*a(1)*tadi1*(akk-1.0)
dyda(4) = dyda(4) + amol*e1(i-k+1)*tpc1*a(3)*tedi1*(akk-1.0)
dyda(6) = dyda(6) + amol*e1(i-k+1)*tpc1*a(5)*tidi1*(akk-1.0)

tadi1 = tadi1*tad1
tedi1 = tedi1*ted1
tidi1 = tidi1*tid1
todi1 = todi1*tod1
```

```
20  continue

25  continue

    dyda(1) = 2.0*sqrt(a(1))*ymod1/a(1)
    dyda(2) = dyda(2)*tpc1/(a(2)*a(2))
    dyda(3) = 2.0*sqrt(a(3))*ymod2/a(3)
    dyda(4) = dyda(4)*tpc1/(a(4)*a(4))
    dyda(5) = 2.0*sqrt(a(5))*ymod3/a(5)
    dyda(6) = dyda(6)*tpc1/(a(6)*a(6))
    dyda(7) = 2.0*sqrt(a(7))*ymod4/a(7)
    dyda(8) = 0.0
    dyda(9) = 0.0
    dyda(10) = 0.0
    dyda(11) = 0.0
    dyda(12) = 0.0
    dyda(13) = 0.0
    dyda(14) = e1(i)
    dyda(15) = 0.0
    dyda(16) = 2.0*sqrt(a(16))
    dyda(17) = 0.0

    ymod = ymod1 + ymod2 + ymod3 + ymod4 + a(14)*e1(i)
     & + a(16)

c   write (*,*) i,ymod

c   write (*,*) dyda(1),dyda(2),dyda(3),dyda(4),dyda(5)

c   do 111 ik=1,ma
c111   write (*,*) 'a(',ik,' = ',a(ik)

    a(1) = sqrt(a(1))
    a(3) = sqrt(a(3))
    a(5) = sqrt(a(5))
    a(7) = sqrt(a(7))
    a(8) = sqrt(a(8))
    a(9) = sqrt(a(9))
    a(11) = sqrt(a(11))
    a(12) = sqrt(a(12))
    a(16) = sqrt(a(16))
    a(17) = sqrt(a(17))

    return
```

```
      end


      subroutine foncs2(i,a,ymod,dyda,ma,ndata2,e2,tpc2,taum)

      implicit double precision (a-h,o-z)

      real*8 a(ma),e2(5000),dyda(ma)
      real*8 tad1(3),tadi1(3),aa1(3),aa2(3),aa3(3),aa4(3),aa5(3),aa6(3)
     & ,ymod1(3),ymod2(3)
      real*8 tedi1,ted1,tadi1,tad1,tidi1,tid1,amol,tpc2,ymod,ymod1,
     & ymod2,ymod3,ymod4,taum,aa1,aa2,aa3,aa4,aa5,aa6, akk


      a(1) = a(1)*a(1)
      a(3) = a(3)*a(3)
      a(5) = a(5)*a(5)
      a(7) = a(7)*a(7)
      a(8) = a(8)*a(8)
      a(9) = a(9)*a(9)
      a(11) = a(11)*a(11)
      a(12) = a(12)*a(12)
      a(16) = a(16)*a(16)
      a(17) = a(17)*a(17)

      do 5 k=1,ma
      dyda(k) = 0.
5     continue


      do 100 id=1,3

      ymod1(id) = 0.0
      ymod2(id) = 0.0

100   continue

      ymod3 = 0.0
      ymod4 = 0.0

      if (i.eq.1) goto 25

      do 105 id=1,3
```

```
      kd = 2*id

      tad1(id) = dexp(-tpc2/a(kd))
      tadi1(id) = 1.0

105   continue

      tid1 = dexp(-tpc2/a(10))
      tidi1 = 1.0

      tod1 = dexp(-tpc2/a(13))
      todi1 = 1.0

      do 110 id=1,3

      kd = 2*id

      aa1(id) = (1.0/a(kd)-1.0/taum)/(1.0/a(kd)-1.0/a(10))
      aa2(id) = (1.0/a(kd)-1.0/taum)/(1.0/a(kd)-1.0/a(13))

      aa3(id) = (1.0/taum-1.0/a(10))/((1.0/a(kd)-1.0/a(10))
     & *(1.0/a(kd)-1.0/a(10)))
      aa4(id) = (1.0/taum-1.0/a(13))/((1.0/a(kd)-1.0/a(13))
     & *(1.0/a(kd)-1.0/a(13)))

      aa5(id) = (1.0/a(kd)-1.0/taum)/((1.0/a(kd)-1.0/a(10))
     & *(1.0/a(kd)-1.0/a(10)))
      aa6(id) = (1.0/a(kd)-1.0/taum)/((1.0/a(kd)-1.0/a(13))
     & *(1.0/a(kd)-1.0/a(13)))

110   continue

      do 20 k=1,i

      akk = k

      if((k.eq.1).or.(k.eq.i)) then
      amol = 0.5
      else
      amol = 1.0
      endif

      do 115 id=1,3
      kd = 1 + 2*(id-1)
```

```
      ymod1(id) = ymod1(id) + amol*e2(i-k+1)*tpc2*
     & a(9)*a(kd)*aa1(id)*
     & (tidi1-tadi1(id))
      ymod2(id) = ymod2(id) + amol*e2(i-k+1)*tpc2*
     & a(12)*a(kd)*aa2(id)*
     & (todi1-tadi1(id))

 115 continue

      ymod3 = ymod3 + amol*e2(i-k+1)*tpc2*a(8)*tidi1
      ymod4 = ymod4 + amol*e2(i-k+1)*tpc2*a(11)*todi1

      do 120 id=1,3
      kd = 2*id
      kkd = 1+2*(id-1)

      dyda(kd) = dyda(kd) + amol*e2(i-k+1)*tpc2*
     & ( a(9)*a(kkd)*(tadi1(id)*(-aa1(id)*tpc2*(akk-1.0) +
     &   aa3(id))-aa3(id)*tidi1) +
     &   a(12)*a(kkd)*(tadi1(id)*(-aa2(id)*tpc2*(akk-1.0) +
     &   aa4(id))-aa4(id)*todi1) )
      dyda(10) = dyda(10) + amol*e2(i-k+1)*tpc2*
     & ( a(9)*a(kkd)*aa1(id)*tidi1*tpc2*(akk-1.0)
     & - aa5(id)*a(9)*a(kkd)*(tidi1-tadi1(id)) )
      dyda(13) = dyda(13) + amol*e2(i-k+1)*tpc2*
     & ( a(12)*a(kkd)*aa2(id)*todi1*tpc2*(akk-1.0)
     & - aa6(id)*a(12)*a(kkd)*(todi1-tadi1(id)) )

 120 continue

      dyda(10) = dyda(10) + amol*e2(i-k+1)*tpc2*
     & a(8)*tidi1*(akk-1.0)*tpc2

      dyda(13) = dyda(13) + amol*e2(i-k+1)*tpc2*
     & a(11)*todi1*(akk-1.0)*tpc2

      do 125 id=1,3

      tadi1(id) = tadi1(id)*tad1(id)

 125 continue

      tidi1 = tidi1*tid1
```

```
      todi1 = todi1*tod1

20    continue

25    continue

      dyda(1) = 2.0*sqrt(a(1))*(ymod1(1)+ymod2(1))/a(1)
      dyda(2) = dyda(2)/(a(2)*a(2))
      dyda(3) = 2.0*sqrt(a(3))*(ymod1(2)+ymod2(2))/a(3)
      dyda(4) = dyda(4)/(a(4)*a(4))
      dyda(5) = 2.0*sqrt(a(5))*(ymod1(3)+ymod2(3))/a(5)
      dyda(6) = dyda(6)/(a(6)*a(6))
      dyda(7) = 0.0
      dyda(8) = 2.0*sqrt(a(8))*ymod3/a(8)
      dyda(9) = 2.0*sqrt(a(9))*
     & (ymod1(1)+ymod1(2)+ymod1(3))/a(9)
      dyda(10) = dyda(10)/(a(10)*a(10))
      dyda(11) = 2.0*sqrt(a(11))*ymod4/a(11)
      dyda(12) = 2.0*sqrt(a(12))*
     & (ymod2(1)+ymod2(2)+ymod2(3))/a(12)
      dyda(13) = dyda(13)/(a(13)*a(13))
      dyda(14) = 0.0
      dyda(15) = e2(i)
      dyda(16) = 0.0
      dyda(17) = 2.0*sqrt(a(17))

      ymod = ymod1(1) + ymod1(2) + ymod1(3) +
     &        ymod2(1) + ymod2(2) + ymod2(3) +
     &        ymod3 + ymod4 + a(15)*e2(i) + a(17)


c     write (*,*) i,ymod,ymod1,ymod2,ymod3

c     write (*,*) dyda(1),dyda(2),dyda(3),dyda(4),dyda(5)

c     do 111 ik=1,ma
c111    write (*,*) 'a(',ik,' = ',a(ik)

      a(1) = sqrt(a(1))
      a(3) = sqrt(a(3))
      a(5) = sqrt(a(5))
      a(7) = sqrt(a(7))
      a(8) = sqrt(a(8))
      a(9) = sqrt(a(9))
```

```
      a(11) = sqrt(a(11))
      a(12) = sqrt(a(12))
      a(16) = sqrt(a(16))
      a(17) = sqrt(a(17))

      return

      end

      subroutine mrqmin(taum,y1,y2,ndata1,ndata2,a,ma,lista,mfit,
     & covar,alpha,nca,chisq,alamda,nstart1,nstart2,e1,e2,itera,
     & da,beta,dyda,tpc1,tpc2,f)

      implicit double precision (a-h,o-z)

      parameter (mmax=20)

      dimension
y1(ndata1),y2(ndata2),a(ma),lista(ma),e1(ndata1),e2(ndata2),
     &  covar(mfit,mfit),alpha(mfit,mfit),atry(mmax),beta(mfit),da(mfit)
     &  ,dyda(mfit),nstart1(3),nstart2(3),f(5000)

      real*8 taum,chisq,alamda,tpc1,tpc2

      save ochisq

c     write (*,*) 'we are in mrqmin. taum = ',taum
c     write (*,*) 'we are in mrqmin. tpc2 = ',tpc2

      if (alamda.lt.0) then
         kk = mfit+1
```

```
      do 12 j=1,ma
         ihit=0
         do 11 k=1,mfit
            if(lista(k).eq.j) ihit=ihit+1
11       continue
         if(ihit.eq.0) then
            lista(kk)=j
            kk = kk+1
         else if (ihit.gt.1) then
            pause 'improper permutation in lista'
         endif
12    continue
   if(kk.ne.(ma+1)) pause 'improper permutation in lista'
   alamda = 0.001

   call mrqcof(y1,y2,ndata1,ndata2,a,ma,lista,mfit,alpha,beta,nca,chisq
    &  ,nstart1,nstart2,e1,e2,tpc1,tpc2,dyda,taum,f)

c  write(*,43) itera,a(1),a(2),chisq/(ndata1+ndata2-nstart1-nstart2-
mfit)
c43 format(I5,5H    ,3(3H   ,F10.3,7H      ))


   do 13 j=1,ma
      atry(j)=a(j)
13 continue
   endif
   ochisq = chisq
   itera = itera+1


   do 15 j=1,mfit
      do 14 k=1,mfit
         covar(j,k)=alpha(j,k)
14    continue
   covar(j,j)=alpha(j,j)*(1.+alamda)
   da(j) = beta(j)
15 continue

c  write (*,*) 'Just before Gaussj.'

   call gaussj(covar,mfit,nca,da,1,1)

c  write (*,*) 'Just after Gaussj.'
```

```
      if(alamda.eq.0) then
         call covsrt(covar,nca,ma,lista,mfit)
         return
      endif
      do 16 j=1,mfit
         atry(lista(j)) = a(lista(j))+da(j)
16    continue


      call
mrqcof(y1,y2,ndata1,ndata2,atry,ma,lista,mfit,covar,da,nca,chisq
     &  ,nstart1,nstart2,e1,e2,tpc1,tpc2,dyda,taum,f)


      if (chisq.lt.ochisq) then
      alamda = 0.1*alamda
      ochisq=chisq
      do 18 j=1,mfit
         do 17 k=1,mfit
            alpha(j,k)=covar(j,k)
17       continue
      beta(j)=da(j)
      a(lista(j))=atry(lista(j))
18    continue
      else
      alamda = 10.*alamda
      chisq=ochisq
      endif
      return
      end


      subroutine mrqcof(y1,y2,ndata1,ndata2,a,ma,lista,mfit,
     & alpha,beta,nalp,chisq
     &  ,nstart1,nstart2,e1,e2,tpc1,tpc2,dyda,taum,f)

      implicit double precision (a-h,o-z)

      dimension y1(5000),y2(5000),alpha(nalp,nalp),beta(mfit),
     &  dyda(mfit),lista(mfit),a(ma),e1(5000),e2(5000)
     & ,nstart1(3),nstart2(3),f(5000)
      real*8 taum,chisq,tpc1,tpc2
```

```
c   write (*,*) 'we are in mrqcof. taum = ',taum
c   write (*,*) 'we are in mrqcof. tpc2 = ',tpc2

    do 112 j=1,mfit
       do 111 k=1,j
       alpha(j,k) = 0.
111    continue
    beta(j) = 0.
112 continue
    chisq=0.

120 do 115 i=nstart1(3),ndata1

       call foncs1(i,a,ymod,dyda,ma,ndata1,e1,tpc1,taum,f)

c      if (i.lt.nstart1) goto 115
       sig2i = 1./y1(i)
       dy = y1(i)-ymod
       do 114 j=1,mfit
          wt=dyda(lista(j))*sig2i
             do 113 k=1,j
             alpha(j,k)=alpha(j,k)+wt*dyda(lista(k))
113          continue
       beta(j)=beta(j)+dy*wt
114    continue
    chisq=chisq+dy*dy*sig2i
115 continue

320 do 315 i=nstart1(1),nstart1(2)

       call foncs1(i,a,ymod,dyda,ma,ndata1,e1,tpc1,taum,f)

c      if (i.lt.nstart1) goto 115
       sig2i = 1./y1(i)
       dy = y1(i)-ymod
       do 314 j=1,mfit
          wt=dyda(lista(j))*sig2i
             do 313 k=1,j
             alpha(j,k)=alpha(j,k)+wt*dyda(lista(k))
313          continue
       beta(j)=beta(j)+dy*wt
314    continue
    chisq=chisq+dy*dy*sig2i
```

```
315 continue

c   write (*,*) 'Chisq value after monomer: ',chisq

220 do 215 i=nstart2(3),ndata2

       call foncs2(i,a,ymod,dyda,ma,ndata2,e2,tpc2,taum)

c      if (i.lt.nstart2) goto 215
       sig2i = 1./y2(i)
       dy = y2(i)-ymod
       do 214 j=1,mfit
          wt=dyda(lista(j))*sig2i
             do 213 k=1,j
             alpha(j,k)=alpha(j,k)+wt*dyda(lista(k))
213          continue
       beta(j)=beta(j)+dy*wt
214    continue
    chisq=chisq+dy*dy*sig2i
215 continue

420 do 415 i=nstart2(1),nstart2(2)

       call foncs2(i,a,ymod,dyda,ma,ndata2,e2,tpc2,taum)

c      if (i.lt.nstart2) goto 215
       sig2i = 1./y2(i)
       dy = y2(i)-ymod
       do 414 j=1,mfit
          wt=dyda(lista(j))*sig2i
             do 413 k=1,j
             alpha(j,k)=alpha(j,k)+wt*dyda(lista(k))
413          continue
       beta(j)=beta(j)+dy*wt
414    continue
    chisq=chisq+dy*dy*sig2i
415 continue

c   write (*,*) 'Chisq value after excimer: ',chisq


    do 117 j=2,mfit
       do 116 k=1,j-1
       alpha(k,j)=alpha(j,k)
```

```
116    continue
117 continue
    return
    end




    subroutine covsrt(covar,ncvm,ma,lista,mfit)

    implicit double precision (a-h,o-z)

    dimension covar(ncvm,ncvm),lista(mfit)

    do 212 j=1,ma-1
       do 211 i=j+1,ma
       covar(i,j) = 0.
211    continue
212 continue
    do 214 i=1,mfit-1
       do 213 j=i+1,mfit
          if(lista(j).gt.lista(i)) then
             covar(lista(j),lista(i))=covar(i,j)
          else
             covar(lista(i),lista(j))=covar(i,j)
          endif
213    continue
214 continue
    swap=covar(1,1)
    do 215 j=1,ma
    covar(1,j) = covar(j,j)
    covar(j,j) = 0.
215 continue
    covar(lista(1),lista(1))=swap
    do 216 j=2,mfit
    covar(lista(j),lista(j))=covar(1,j)
216 continue
    do 218 j=2,ma
       do 217 i=1,j-1
       covar(i,j)=covar(j,i)
217 continue
218 continue
```

```
      return
      end




      subroutine gaussj(a,n,np,b,m,mp)

      implicit double precision (a-h,o-z)

      parameter (nmax=50)
      dimension a(np,np),b(np),ipiv(nmax),indxr(nmax),indxc(nmax)

      do 311 j=1,n
         ipiv(j) = 0
311   continue

      do 322 i=1,n
         big = 0.

         do 313 j=1,n

            if (ipiv(j).ne.1) then

            do 312 k=1,n

            if(ipiv(k).eq.0) then

               if (abs(a(j,k)).ge.big) then
               big = abs(a(j,k))
               irow = j
               icol = k
               endif

               else if (ipiv(k).gt.1) then
               pause 'singular matrix'
               endif

312         continue

            endif
```

```
313    continue

   ipiv(icol) = ipiv(icol) + 1

   if (irow.ne.icol) then

      do 314 l=1,n
         dum = a(irow,l)
         a(irow,l) = a(icol,l)
         a(icol,l) = dum
314    continue

         dum = b(irow)
         b(irow) = b(icol)
         b(icol) = dum

   endif

   indxr(i) = irow
   indxc(i) = icol

   if (a(icol,icol).eq.0)  pause 'singular matrix'

   pivinv = 1./a(icol,icol)

   a(icol,icol) = 1.

   do 316 l=1,n
      a(icol,l) = a(icol,l)*pivinv
316 continue

      b(icol) = b(icol)*pivinv

   do 321 ll=1,n
      if (ll.ne.icol) then

         dum = a(ll,icol)
         a(ll,icol) = 0.

         do 318 l=1,n
            a(ll,l) = a(ll,l) - a(icol,l)*dum
318       continue
```

```
             b(ll) = b(ll) - b(icol)*dum

          endif

321  continue

322  continue

       do 324 l=n,1,-1
          if (indxr(l).ne.indxc(l)) then

             do 323 k=1,n
                dum = a(k,indxr(l))
                a(k,indxr(l)) = a(k,indxc(l))
                a(k,indxc(l)) = dum
323          continue

          endif

324  continue

       return
       end
```

Legends for data S1 to S3

Data S1: PyCX-PAMAM-G0 0~615mM NaCl SteadyStateFluorescence data

Data (wavelength, fluo. int.) for the fluorescence spectra of the PyCX-PAMAM-G0 dendrimers acquired with the 50 mM SDS solution with different NaCl concentrations.

Data S2: PyCX-PAMAM-G0 0~615mM NaCl TimeResolvedFluorescence data

Brief caption: Instrument response functions and fluorescence decays of the PyCX-PAMAM-G0 dendrimers acquired with the 50 mM SDS solution with different NaCl concentrations.

Data S3: PyCX-PAMAM-G0 0~615mM NaCl UV-vis data

Brief caption: Data (wavelength, fluo. int.) for the absorption spectra of the PyCX-PAMAM-G0 dendrimers acquired with the 50 mM SDS solution with different NaCl concentrations.
